# Formulation and Investigation of the In Vitro Immunostimulatory Potential of Microparticulate Guanine-α-D-Fructose in Vaccine Candidates

**DOI:** 10.3390/vaccines14090724

**Published:** 2026-08-22

**Authors:** Yashkumar Harsoda, Mahek Gulani, Snehitha Akkineni, Aditi Satoskar, Amarae Ferguson, Tanisha Manoj Arte, Mohammad N. Uddin, Christiane Chbib, Martin J. D’Souza

**Affiliations:** 1 Vaccine Nanotechnology Laboratory, College of Pharmacy, Center for Drug Delivery Research, Mercer University, Atlanta, GA 30341, USA; yashkumar.pankajbhai.harsoda@live.mercer.edu (Y.H.); mahekanil.gulani@live.mercer.edu (M.G.); snehitha.akkineni@live.mercer.edu (S.A.); aditi.suchitkumar.satoskar@live.mercer.edu (A.S.); amarae.ferguson@live.mercer.edu (A.F.); tanisha.manoj.arte@live.mercer.edu (T.M.A.); uddin_mn@mercer.edu (M.N.U.); 2 Dr. Kiran C. Patel College of Allopathic Medicine (NSU-MD), Nova Southeastern University, Fort Lauderdale, FL 33328, USA; cchbib@nova.edu

**Keywords:** immunostimulatory compound, flow cytometry, MHC I, MHC II, vaccines, PLGA microparticles, Guanine-α-D-Fructose

## Abstract

Background/Objectives: Particulate vaccine formulations require delivery systems and immunostimulatory components that support antigen-presenting cell interaction while maintaining formulation stability and cytocompatibility. This study examines Guanine-α-D-Fructose (GDF), a guanine–fructose small-molecule immunostimulatory candidate, incorporated into poly(lactic acid-co-glycolic acid) (PLGA) microparticles and benchmarked in vitro against established adjuvant formulations containing Alum or AddaVax. Methods: GDF-loaded microparticles were prepared and characterized for particle size, morphology, surface charge, entrapment efficiency, and release behavior. Their in vitro immunostimulatory activity was evaluated using murine dendritic cells. To explore formulation compatibility, GDF MPs were tested in combination with multiple particulate antigen formulations, including measles, gonorrhea, SARS-CoV-2, influenza A (H3N2), and Zika. MTT (3-(4,5-dimethylthiazol-2-yl)-2,5-diphenyltetrazolium bromide) assay was utilized to measure cell viability, while nitric oxide generation was measured with the Griess assay. Dendritic cell activation-associated surface marker expression was analyzed by flow cytometry using MHC I, MHC II, CD80, and CD40. Results: The particles showed consistent spherical morphology, sustained GDF release, and favorable cytocompatibility at concentrations up to 250 µg/mL, with reduced viability at higher concentrations. GDF MPs increased nitric oxide production and activation-associated marker expression, with responses benchmarked against Alum and AddaVax and comparable in several assay conditions. Increased autophagosome-associated fluorescence was also observed, suggesting modulation of autophagy-related cellular activity; however, direct antigen-processing or antigen-presentation assays are still required. Conclusions: Overall, these findings support GDF-loaded PLGA microparticles as an in vitro immunostimulatory particulate formulation suitable for further mechanistic and in vivo evaluation in vaccine-related systems.

## 1. Introduction

One of the best public health strategies for preventing and controlling infectious diseases is still vaccination. While advances in antigen discovery and recombinant technologies have significantly improved vaccine specificity and safety, many modern antigens, particularly purified subunit and synthetic antigen exhibits limited intrinsic immunogenicity. As a result, adjuvants are commonly incorporated to strengthen and shape immune responses. Adjuvants function by enhancing antigen presentation, promoting innate immune activation, and shaping the magnitude and quality of downstream adaptive immune responses [1,2,3]. Despite their critical role, the number of adjuvants approved for human use remains limited, highlighting the continued need for safe, effective, and versatile immunostimulatory formulation strategies. Conventional adjuvants such as aluminum salts (Alum) have been extensively utilized for decades due to their capacity to boost humoral immunity and established safety profile. However, Alum is known to be a weak inducer of cellular immunity and exhibits limited capacity to promote dendritic cell activation and Th1-type immune responses. More recently developed oil-in-water emulsions, such as AddaVax, have demonstrated improved immunostimulatory properties by enhancing antigen uptake and local immune cell recruitment [4,5]. Nevertheless, these systems still face challenges related to formulation stability, reactogenicity, and inconsistent activation of antigen-presenting cells. These limitations underscore the importance of identifying immunostimulatory molecules and adjuvant candidates that can stimulate innate immune pathways while maintaining high cell viability.

In parallel with immunostimulatory compounds, delivery systems have emerged as a critical component of modern formulation design. Microparticle-based carriers can protect encapsulated molecules, enhance cellular uptake, enable sustained release, and support antigen-presenting-cell interaction and intracellular processing. Among these carriers, poly(lactic acid-co-glycolic acid) (PLGA) has gained notable focus because of its biodegradability, cytocompatibility, and regulatory approval for human use [6]. PLGA-based microparticles are generally readily internalized into antigen-presenting cells, particularly dendritic cells, supporting intracellular delivery and processing of associated immunostimulatory agents. Furthermore, PLGA enables controlled-release kinetics, which can prolong local compound availability and reduce the need for repeated dosing. These properties make PLGA an attractive framework for vaccine-related particulate formulation design [7].

Particle size is an important design parameter in PLGA-based vaccine-related particulate delivery systems because it can influence particle trafficking, antigen-presenting cell interaction, intracellular processing, and release behavior. Smaller particles have been shown to drain more readily to lymph nodes, whereas larger particles in the low-micron range may depend more on antigen-presenting cell-associated transport and uptake [8]. Particle size and surface charge can also affect uptake by human dendritic cells, supporting the importance of controlling these physicochemical properties during particulate vaccine design [9]. In PLGA-based vaccine systems, particle size has been shown to influence dendritic cell internalization, MHC I and co-stimulatory marker expression, antigen-specific CD8^+^ T-cell responses, and IgG subtype profiles [10]. In addition, PLGA microparticulate vaccine systems have been reported to induce strong antibody responses in vivo, supporting the continued relevance of micron-sized polymeric particles for vaccine delivery [11]. Together, these studies indicate that particle size can influence downstream immune responses by affecting how particulate vaccine systems are trafficked, internalized, processed, and retained over time.

The rational design of immunostimulatory molecules that combine biological activity with favorable physicochemical properties represents an emerging strategy in vaccine-related formulation research. Nucleobase-derived structures, in particular, have attracted interest due to their potential to interact with innate immune sensors and influence antigen-presenting cell function. Certain guanine-containing nucleoside analogs and guanine-rich oligonucleotide structures have been reported to modulate innate immune sensing pathways. The direct application of such compounds is often hindered by low solubility or instability [12,13]. In this context, Guanine-α-D-Fructose (GDF) was designed as a small-molecule immunostimulatory candidate integrating multiple functional features within a single molecular framework. The guanine moiety was incorporated to provide immunostimulatory potential, while the fructose ring was introduced to enhance aqueous solubility and biological compatibility. The glycosidic linkage was incorporated as part of the intended molecular scaffold. To our knowledge, GDF has not been investigated in prior studies as an immunomodulatory molecule in particulate vaccine-related formulations, making it a novel candidate for systematic evaluation [14,15].

In the present study, GDF was formulated into PLGA-based MPs to evaluate its delivery characteristics and in vitro immunostimulatory activity. The microparticles were physicochemically characterized and evaluated for cytocompatibility and activation-associated responses using murine dendritic cells in vitro. The formulation compatibility of GDF MPs was further assessed in combination with multiple viral and bacterial particulate antigen formulations, such as measles, gonorrhea, SARS-CoV-2 Omicron strain, influenza A (H3N2 strain), and Zika, in order to examine their performance across multiple antigen contexts. A comprehensive panel of assays was employed to assess cytotoxicity, nitric oxide production, and dendritic cell activation-associated surface markers including MHC class I, MHC class II, CD40, and CD80. The performance of GDF MPs was directly compared with established adjuvants, including Alum and AddaVax, to benchmark early in vitro immunostimulatory readouts [16,17,18].

By integrating a small-molecule immunostimulatory candidate with a well-established biodegradable delivery system, this work seeks to support the formulation-level evaluation of GDF-loaded PLGA microparticles while maintaining a favorable cytocompatibility profile [19]. The findings presented here provide foundational in vitro evidence supporting the potential of GDF-loaded PLGA microparticles as an immunostimulatory particulate formulation for future vaccine-related applications. We hypothesize that GDF-loaded PLGA microparticles increase dendritic cell activation-associated marker expression and nitric oxide production under in vitro conditions, with responses benchmarked against established adjuvants.

## 2. Materials and Methods

### 2.1. Materials

Dichloromethane, poly(vinyl alcohol) (PVA), and trehalose used for microparticle fabrication were purchased from Sigma–Aldrich (Burlington, MA, USA). The biodegradable poly(lactic acid-co-glycolic acid)(PLGA; 75:25) was obtained from Evonik Industries (Essen, Germany). The immunostimulatory compound Guanine-α-D-Fructose (GDF) used in this study was originally obtained commercially from MuseChem (Paoli, PA, USA), provided as a gift by Dr. Christiane Chbib, and used as supplied for the preparation of GDF-loaded PLGA microparticles. Bovine serum albumin (BSA) was obtained from Fisher Scientific (Waltham, MA, USA). The murine dendritic cell line DC2.4 was provided by Dr. Rock (Dana-Farber Cancer Institute, Boston, MA, USA). Cell culture reagents including Dulbecco’s Modified Eagle’s Medium, fetal bovine serum, penicillin, and streptomycin were obtained from ATCC (Manassas, VA, USA). Commercial adjuvants aluminum hydroxide (Alum) and AddaVax (a squalene-based oil-in-water nanoemulsion adjuvant) were purchased from InvivoGen (San Diego, CA, USA). Viral antigens for the whole inactivated virus of SARS-CoV-2 and influenza A BPL-inactivated whole virus (H3N2) were obtained from BEI Resources (Manassas, VA, USA). The attenuated Measles virus Edmonston-Zagreb strain was acquired from the Serum Institute of India (Pune, India). The *Neisseria gonorrhoeae* bacteria (whole-cell inactivated *N. gonorrhea* vaccine antigen from the strain CDC-F62) was provided by Dr. Shafer (Emory University, Atlanta, GA, USA), and the Zika virus strain PRVABC59 was obtained from the Centers for Disease Control and Prevention (CDC, Atlanta, GA, USA). Reagents used in nitric oxide quantification, including sodium nitrite, sulfanilamide, and N-(1-naphthyl)ethylenediamine dihydrochloride, were purchased from Thermo Fisher Scientific (Rockford, IL, USA). The reagent (3-(4,5-dimethylthiazol-2-yl)-2,5-diphenyl tetrazolium bromide) was obtained from Sigma–Aldrich (St. Louis, MO, USA). Antibodies used for flow cytometric analysis were purchased from eBioscience (San Diego, CA, USA) and BioLegend (San Diego, CA, USA).

### 2.2. Methods

#### 2.2.1. Double-Emulsion Formulation of Viral Vaccine and Adjuvant Microparticles

**Figure 1:**
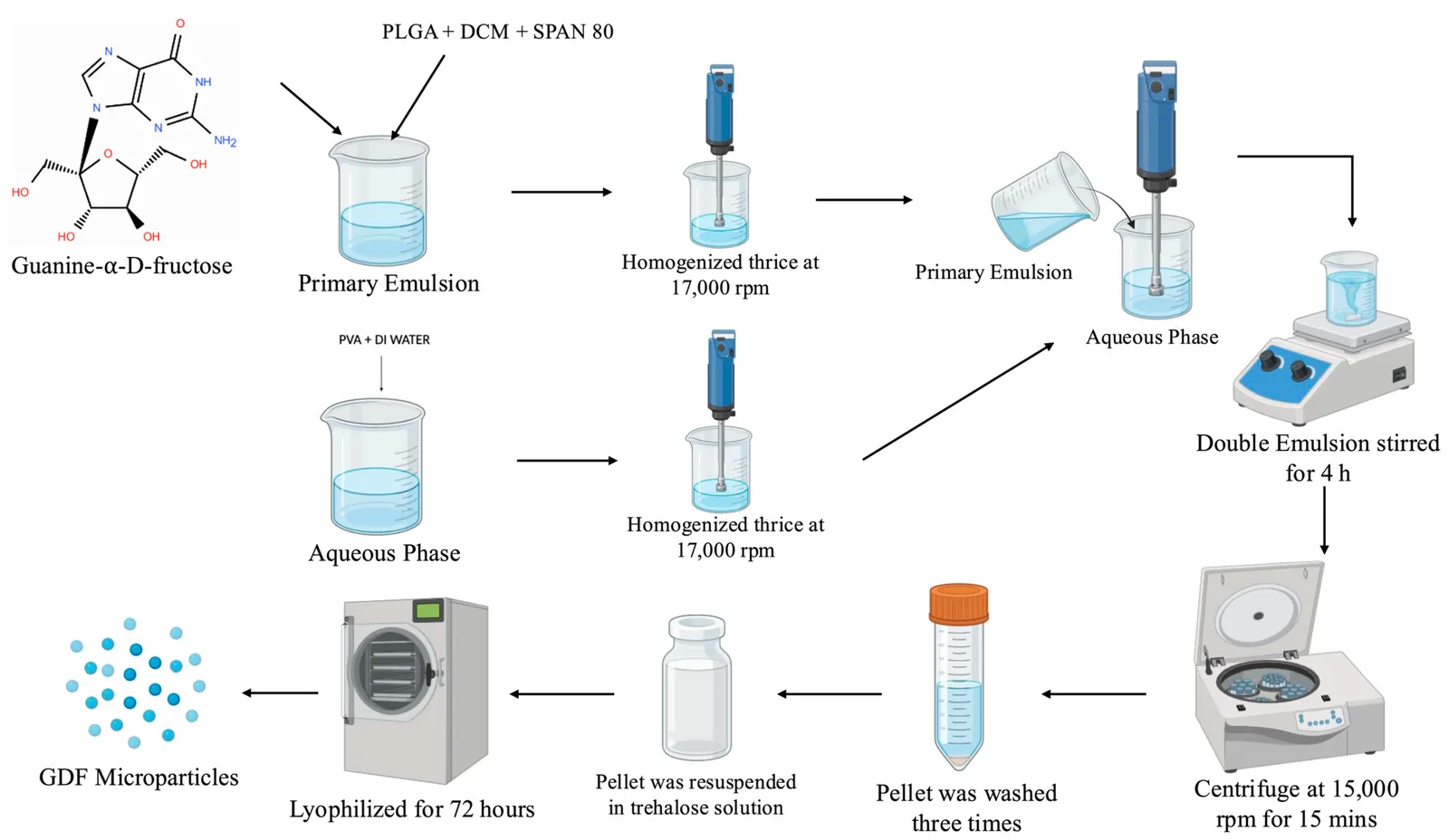
Schematic representation of GDF microparticle preparation using the double emulsion solvent evaporation method (W/O/W).

Microparticles were made using a two-step emulsion solvent removal (w/o/w) process, a method previously validated for the encapsulation of vaccine antigens and adjuvants within PLGA-based carriers. This formulation approach was used to make microparticles encapsulating vaccine antigens derived from Influenza A (H3N2), Zika virus, SARS-CoV-2 Omicron strain, adjuvants (Alum, AddaVax) and GDF [20]. Each antigen and adjuvant was formulated separately into microparticles. For the combination groups, the independently prepared microparticle suspensions were mixed at the doses listed in Table 1 immediately before treatment of the DC2.4 cells. GDF and the vaccine antigens were therefore administered as separate microparticle formulations and were not co-encapsulated within the same particles. In brief, the antigen was prepared in phosphate-buffered saline (PBS, pH 7.4) and introduced into a solution of PLGA in dichloromethane (DCM). The mixture was homogenized at 17,000 rpm using a probe homogenizer, with alternating 30 s on/off pulses for a total duration of 2 min, to generate the primary water-in-oil (W_1_/O) emulsion. The resulting primary emulsion was subsequently added to an aqueous poly(vinyl alcohol) (PVA) solution (0.1% *w/v*) and homogenized at 17,000 rpm for 2 min to generate the water-in-oil-in-water (W_1_/O/W_2_) double emulsion (Figure 1). The resulting emulsion was stirred magnetically at room temperature for 4 h to allow for complete solvent removal. Following solvent removal, the emulsion was centrifuged at 15,000 rpm for 15 min at 15 °C to obtain the microparticles. The pellet was washed and resuspended in a cryoprotectant solution containing trehalose (20 mg in 1 mL DI water), which has been shown to preserve particle morphology and prevent aggregation during freeze-drying [21]. After a second centrifugation step, the pellet was resuspended in the remaining trehalose solution, transferred into pre-weighed vials, and reweighed to determine formulation yield. Samples were lyophilized for 72 h to obtain a dry, free-flowing microparticle powder suitable for long-term in vitro studies. This formulation strategy has previously been shown to produce spherical PLGA microparticles with controlled size distribution, high recovery yield, and reproducible encapsulation efficiency, while maintaining biological compatibility with antigen-presenting cells [20]. Entrapment efficiency of GDF-loaded PLGA microparticles was estimated by quantifying free, unencapsulated GDF following centrifugal ultrafiltration. Briefly, GDF-loaded microparticles were suspended in phosphate-buffered saline (PBS) at a concentration of 1 mg/mL and subjected to ultrafiltration using Vivaspin centrifugal filter units. The ultrafiltration membrane retained the microparticle fraction while permitting separation of free GDF present in the aqueous phase. The absorbance of the collected filtrate was measured at 253 nm using a UV–Vis spectrophotometer, and the concentration of free GDF was calculated using a previously established GDF calibration curve. Entrapment efficiency was estimated using the following equation:

(1) $$Entrapment\;Efficiency=\frac{\left(Theoretical\;GDF\;content-Free\;GDF\;in\;the\;Filtrate\right)\times 100}{Theoretical\;GDF\;content}$$

where free GDF represents the amount of unencapsulated GDF detected in the filtrate and theoretical GDF content represents the expected GDF amount based on the formulation loading.

#### 2.2.2. Preparation of Spray-Dried Microparticles Containing Inactivated *N. gonorrhoeae* Whole-Cell Antigen and Viral Measles Vaccine

Biodegradable vaccine microparticles were generated through spray drying using a Büchi Mini Spray Dryer. This approach was applied to produce microparticles incorporating formalin-neutralized whole-cell *Neisseria gonorrhoeae* antigen [22]. To obtain formulations containing 10% antigen loading, the carrier matrix was first prepared by dissolving 360 mg bovine serum albumin (BSA) in 20 mL of water. BSA served as the albumin polymeric biodegradable scaffold, functioning similarly to a protein carrier used in antigen conjugation strategies [23]. Because antigen-presenting cells possess albumin-binding receptors, incorporation of the antigen within the BSA matrix promotes cellular uptake of the particles and facilitates antigen internalization, which can enhance cellular immune responses. After complete dissolution of the BSA, glutaraldehyde was introduced at a proportion of 400 µL for each 2 g of BSA to initiate controlled intermolecular crosslinking of the protein matrix. The mixture was then stirred overnight at 300 rpm at room temperature in the dark. Once the crosslinking step was complete, 40 mg of sodium bisulfite was added to quench and inactivate any residual glutaraldehyde remaining in the solution. The pre-crosslinked BSA preparation generated during the overnight reaction was subsequently combined with the inactivated antigen. From this resulting formulation, 100 mg of the mixture was dissolved in 10 mL of deionized water to produce a feed solution suitable for spray drying. The suspension was then processed through the spray dryer using a 0.5 mm atomization nozzle, maintained at a nozzle temperature of −5 °C. Particle formation occurred under the following operating conditions: inlet temperature of 110 °C, feed flow rate of 18 mL h^−1^, and aspirator capacity set to 100%. These conditions yielded dry microparticles. Microparticles containing measles vaccine antigen (Edmonston–Zagreb strain) were produced using identical spray-drying parameters, except that the measles antigen was incorporated at 5% (*w*/*w*) of the formulation.

#### 2.2.3. Comprehensive Physicochemical Characterization of Vaccine Microparticles

To comprehensively evaluate the physical attributes, formulation robustness, and structural integrity of the microparticles, a series of complementary physicochemical characterization techniques was employed. These analyses were designed to sequentially assess the size of the particles, particle population characteristics, surface charge behavior, and morphological features. Recovery yield reflects the proportion of the total formulation mass successfully recovered as dry microparticles and serves as an indicator of batch-to-batch reproducibility and process-related material losses. The mass of the microparticles was analyzed pre-lyophilization and post-lyophilization to achieve an accurate loss of component during the process [24]. The percent recovery yield was calculated using the following Equation (2):

(2) $$Percent\;Recovery\;Yield=\frac{Dry\;Microparticles\;Weight\times 100}{Pre\;Lyophilization\;Weight}$$

Measurements were performed using a Malvern Nano ZS instrument (Luebeck Germany), employing dynamic light scattering for particle size determination and electrophoretic light scattering for zeta potential analysis. For sample preparation, microparticles (10 µg) were dispersed in 2 mL of deionized water to generate a dilute and homogeneous suspension suitable for light-scattering measurements. Suspensions were gently mixed to minimize aggregation prior to analysis, and all measurements were conducted in triplicate under identical conditions to ensure reproducibility and statistical reliability. Finally, surface morphology and structural features of the microparticles were studied using scanning electron microscopy (SEM) to visually corroborate particle formation and assess surface characteristics. Dry microparticle samples were placed onto aluminum stubs using electrically conductive carbon adhesive tabs and later coated with a thin gold layer via sputter deposition to enhance surface conductivity. Gold coating was performed for 30 s at a sputtering current of 50 mA, with chamber pressure maintained at 0.05 mbar and argon gas pressure controlled between 0.15 and 0.2 mbar. Morphological imaging was carried out using a Phenom benchtop scanning electron microscope (Nanoscience Instruments, Phoenix, AZ, USA).

#### 2.2.4. Evaluation of Nitric Oxide Release as a Marker of Innate Immune Activation

Nitric oxide (NO) release was used as an indicator of innate immune activation in response to microparticle treatment. DC2.4 murine dendritic cells were seeded into 96-well plates at 5 × 10^4^ cells per well. The microparticle formulations were suspended in complete cell-culture medium (complete medium consisted of Dulbecco’s Modified Eagle’s Medium supplemented with 10% (*v*/*v*) fetal bovine serum and 1% (*v*/*v*) penicillin–streptomycin) and mixed to obtain uniform suspensions and then added to the cells at the doses specified in Table 1 [25,26]. Blank PLGA-microparticles served as negative controls. Following a 24 h incubation at 37 °C, supernatant from wells were collected and transferred to new 96-well plates for nitrite measurement using the Griess reaction. Equal amounts of 1% sulfanilamide in 5% phosphoric acid and 0.1% N-1-naphthylethylenediamine dihydrochloride in deionized water were added to each well, and the plate was incubated for 10 min under light-protected conditions [27]. Absorbance was recorded at 540 nm using a BioTek Synergy H1 microplate reader (BioTek Instruments, Winooski, VT, USA). Nitrite concentrations were calculated from a sodium nitrite standard curve.

#### 2.2.5. In Vitro Release Study

The release profile of Guanine-α-D-Fructose (GDF) microparticles was studied through an in vitro release experiment to characterize the temporal release behavior of the encapsulated compound [22]. Briefly, 5 mg of GDF MPs, and an equivalent amount of free GDF for the control, were placed in pre-soaked dialysis bags (MWCO 3.5 kDa), sealed, and immersed in 25 mL of phosphate-buffered saline (PBS, pH 7.4) at 37 °C under continuous magnetic stirring at 90 rpm.

At preplanned sampling durations spanning 0–72 h (0, 0.25, 0.5, 1, 2, 3, 6, 12, 24, 36, 48, 60, and 72 h), 0.8 mL aliquots of the release medium were withdrawn for analysis. Following each collection, the sampled volume was immediately substituted with an equivalent volume of PBS to maintain constant sink conditions throughout the experiment. The amount of GDF released into the supernatant was quantified by measuring absorbance at 253 nm using a NanoDrop 2000c UV–Vis spectrophotometer (Thermo Fisher Scientific, Waltham, MA, USA). To further investigate the release mechanism, the experimental data were fitted to several widely used drug-release kinetic models, such as zero-order, first-order [28], Higuchi [29,30], and Korsmeyer–Peppas models [31,32]. Together, these methods allowed for a comprehensive evaluation of the release profile and kinetic behavior of GDF from the microparticles.

#### 2.2.6. In Vitro Cytotoxicity of GDF Microparticles

To evaluate the biocompatibility of the microparticle formulations, the cytotoxic response of DC2.4 was assessed using the MTT assay [33]. Briefly, DC2.4 cells were seeded into 96-well plates at a density of 5 × 10^4^ cells per well, microparticles were suspended in complete cell-culture medium (complete medium consisted of Dulbecco’s Modified Eagle’s Medium supplemented with 10% (*v*/*v*) fetal bovine serum and 1% penicillin–streptomycin (*v*/*v*) and cultured overnight to allow for cell attachment. The following day, cells were exposed to graded concentrations (31.25–1000 μg/mL) of GDF microparticles (GDF MPs). After 24 h of incubation at 37 °C, the medium was carefully aspirated, and cells were treated with MTT solution (10 μg/2 mL in PBS). Plates were kept for 4 h in the dark, allowing viable cells to metabolize MTT into formazan crystals. To solubilize the crystals, dimethyl sulfoxide (DMSO) was added to each well, and the plates were shaken gently at room temperature for 20 min under light-protected conditions. Optical density was then measured at 570 nm using a BioTek Synergy H1 microplate reader (BioTek Instruments, Winooski, VT, USA), and the percentage of the untreated control group was used to express cell viability.

#### 2.2.7. Flow Cytometric Evaluation of Dendritic Cell Activation Marker Expression

To assess dendritic cell activation in response to microparticle formulations, flow cytometry was performed using the DC2.4 cell line. Cells were seeded in 48-well plates and allowed to adhere overnight under standard culture conditions. The subsequent day, cells were treated with treatment and were suspended in complete cell-culture medium (complete medium consisted of Dulbecco’s Modified Eagle’s Medium supplemented with 10% (*v*/*v*) fetal bovine serum and 1% penicillin–streptomycin (*v*/*v*) as mentioned in Table 1). Controls added were untreated and blank microparticle-treated cells. After 24 h incubation, cells were harvested by gentle detachment with cell scrapers and washed four times with PBS. Cells were then stained for 40 min on ice in the dark with FITC-conjugated anti-mouse MHC class I (H-2K^b^; clone AF6-88.5.5.3; mouse IgG2a, κ isotype; eBioscience), FITC-conjugated anti-mouse MHC class II (I-A/I-E; clone M5/114.15.2; rat IgG2b, κ isotype; BioLegend), APC-conjugated anti-mouse CD40 (clone 3/23; rat IgG2a, κ isotype; BioLegend), and APC-conjugated anti-mouse CD80 (clone 16-10A1; Armenian hamster IgG isotype; BioLegend), and at least 5000 events were recorded per sample. Events corresponding to the principal cellular population were selected based on forward- and side-scatter characteristics to exclude debris. Activation was quantified by comparing marker expression levels of treated groups against untreated controls.

#### 2.2.8. Dendritic Cell Autophagic Response to Microparticle Formulations

Autophagy is a central regulatory mechanism in dendritic cells, influencing cellular activation, antigen processing, and cytokine secretion [34]. Antigen presentation via both the MHC class II and MHC class I pathways has been shown to depend on autophagic activity. To investigate the extent to which different formulations modulate autophagosome formation in dendritic cells, intracellular autophagic vesicles were evaluated using complementary qualitative and quantitative approaches [35]. Autophagosome formation was assessed using the CYTO-ID autophagy detection system, applied following the company’s recommendations. Quantitative analysis was performed by flow cytometry, while fluorescence-based live-cell imaging was employed to visualize autophagic structures at the cellular level. For qualitative visualization, dendritic cells were seeded at a density of 5 × 10^4^ cells per well in 24-well culture plates and allowed to adhere overnight. After 24 h, cells were exposed to the designated treatment conditions, including untreated controls, GDF suspension, and GDF-loaded microparticles, using doses corresponding to those specified for each group. All microparticle formulations were suspended in complete cell-culture medium (complete medium consisted of Dulbecco’s Modified Eagle’s Medium supplemented with 10% (*v*/*v*) fetal bovine serum and 1% penicillin–streptomycin (*v*/*v*) before treatment). Treated cells were incubated for an additional 24 h at 37 °C under 5% CO_2_. Following incubation, cells were washed with phosphate-buffered saline and stained with the CYTO-ID autophagy dye in combination with Hoechst 33342 nuclear stain for 30 min at 37 °C. Excess dye was removed, and cells were imaged using a fluorescence microscope equipped with FITC and DAPI filter sets (Lionheart FX, BioTek, VT, USA). Nuclear DNA was visualized via Hoechst staining, while autophagosomes were identified based on CYTO-ID fluorescence. Quantitative evaluation of autophagosome expression was conducted using a parallel staining protocol, with cells labeled exclusively with the CYTO-ID reagent. Treatment groups included untreated cells, GDF solution, GDF-loaded microparticles, Alum-loaded microparticles, gonorrhea microparticles, gonorrhea–Alum microparticles, and gonorrhea–GDF microparticles. After treatment, cells were gently detached by trypsinization, resuspended in appropriate buffer, and analyzed using a BD Accuri C6 Plus flow cytometer (BD Biosciences, San Jose, CA, USA). Autophagic activity was quantified using Mean Fluorescence Intensity (MFI).

#### 2.2.9. Statistical Evaluation of Experimental Data

All statistical evaluations were carried out using GraphPad Prism Version 10.3.1. Experimental data are presented as mean values accompanied by the standard error of the mean (SEM). Unless otherwise stated, each experiment was conducted in *n* = 6. Statistical comparisons among multiple groups were carried out using one-way analysis of variance (ANOVA), followed by Tukey’s multiple-comparisons post hoc test to identify pairwise differences. Levels of statistical significance were denoted as follows: *p* > 0.05 (ns, not significant), *p* ≤ 0.05 (*), *p* ≤ 0.01 (**), *p* ≤ 0.001 (***), and *p* ≤ 0.0001 (****). For all analyses, differences were considered statistically significant when *p* < 0.05.

## 3. Results

### 3.1. Physicochemical Properties and Structural Features of Microparticles

Dynamic light scattering analysis revealed that the GDF-loaded microparticles exhibited a mean hydrodynamic diameter of 1.436 ± 0.049 μm, indicating formation within the submicron–micron size range with good batch uniformity. Measurements of surface charge showed a negative zeta potential of −14.8 ± 0.7 mV.

Entrapment efficiency was estimated by measuring the amount of free GDF recovered in the filtrate following centrifugal ultrafiltration. Based on the theoretical GDF loading of the formulation and the concentration of free GDF detected at 253 nm, the estimated entrapment efficiency of GDF-loaded PLGA microparticles was approximately 86.4%, indicating efficient incorporation of GDF within the PLGA microparticle formulation. The physicochemical characteristics of GDF-loaded PLGA microparticles are summarized in Table 2.

**Figure 2:**
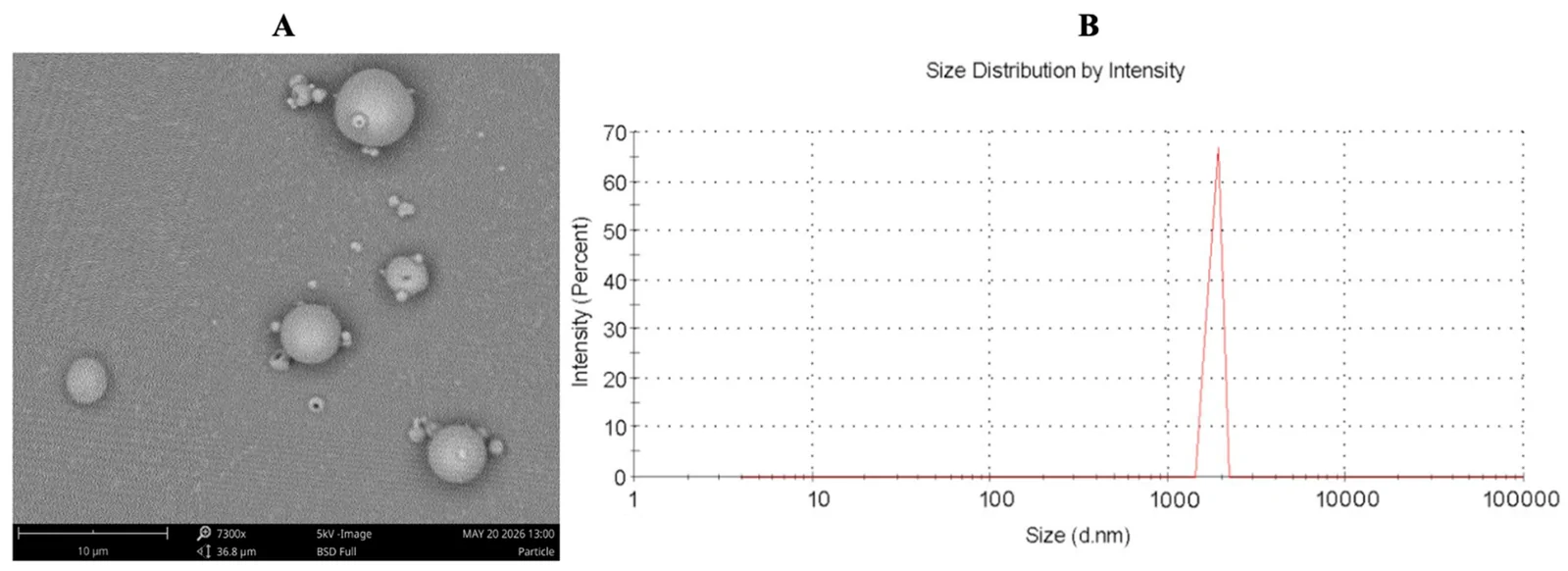
Morphology and particle-size distribution of Guanine-α-D-Fructose-loaded PLGA microparticles. (**A**) Representative scanning electron microscopy (SEM) image acquired at 7300× magnification, showing predominantly spherical microparticles with smooth surface morphology and limited aggregation. (**B**) Intensity-weighted particle-size distribution of GDF-loaded PLGA microparticles measured by dynamic light scattering using a Malvern Zetasizer Nano ZS. The particles exhibited a mean hydrodynamic diameter (Z-average) of 1.436 ± 0.049 µm with a polydispersity index (PDI) of 0.21.

Morphological examination using scanning electron microscopy further supported successful particle fabrication. SEM images showed predominantly spherical microparticles with smooth surface characteristics and no evidence of major structural collapse. Variability in particle diameter was observed, along with the presence of smaller surface-associated satellite particulates, which may have originated during the emulsification and solvent evaporation process and are consistent with the measured polydispersity of the formulation (Figure 2).

### 3.2. In Vitro Evaluation of Nitric Oxide Production Following Microparticle Treatment

**Figure 3:**
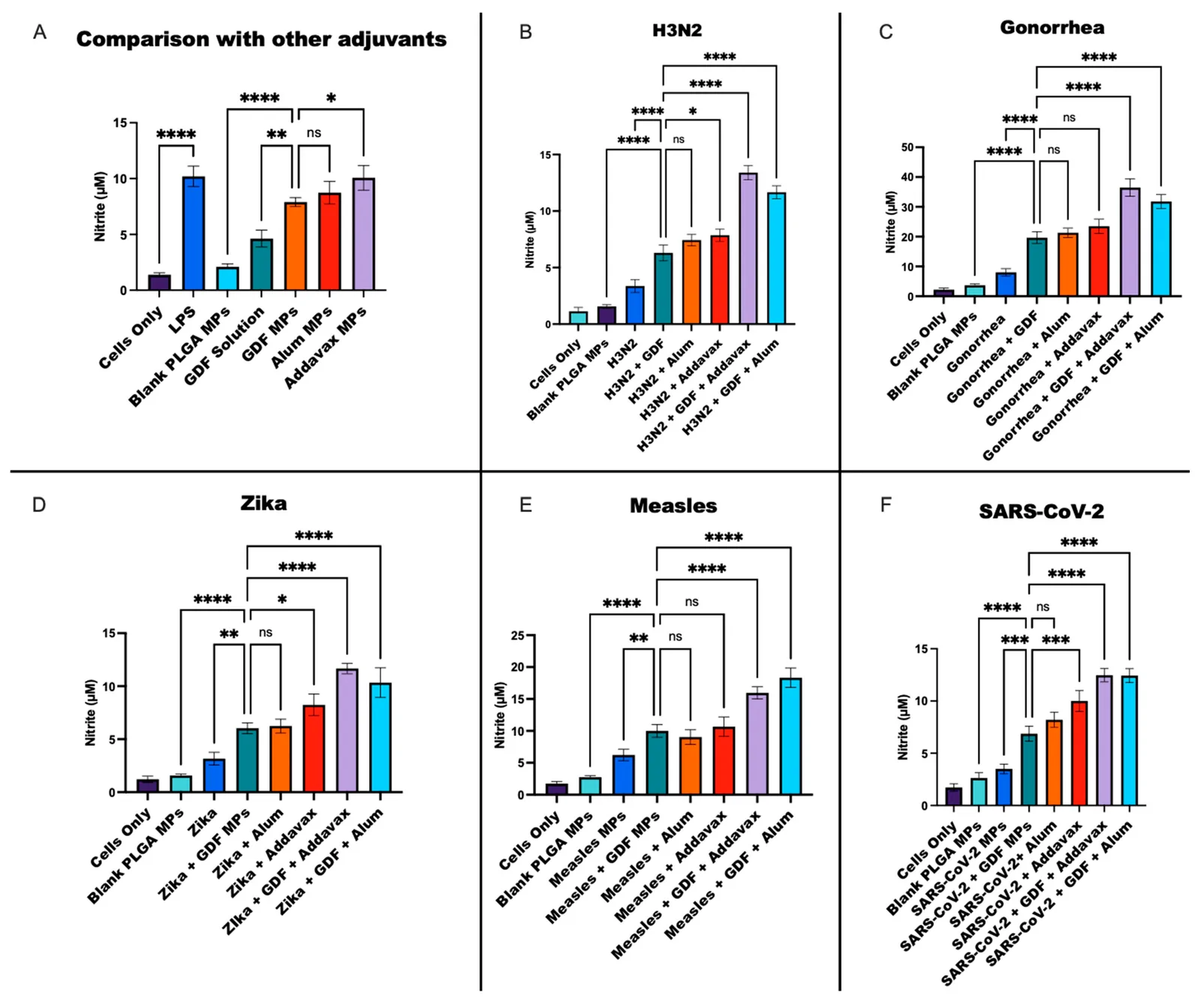
Comparison of nitric oxide production in dendritic cells following stimulation with Guanine-α-D-Fructose microparticle formulation, vaccine microparticles, and other adjuvant microparticles. (**A**) Nitrite production in dendritic cells treated with Blank PLGA microparticles, soluble GDF, GDF-loaded microparticles, Alum microparticles, and AddaVax microparticles, with LPS included as a positive control. (**B**) Nitrite production induced by H3N2 vaccine microparticles alone or in combination with Blank PLGA microparticles, GDF, Alum, or AddaVax formulations. (**C**) Nitrite levels following treatment with gonorrhea vaccine microparticles alone or combined with Blank PLGA microparticles, GDF, Alum, or AddaVax formulations. (**D**) Nitrite production in dendritic cells treated with Zika vaccine microparticles alone or in combination with Blank PLGA microparticles, GDF, Alum, AddaVax, or dual-adjuvant formulations. (**E**) Nitrite production induced by measles vaccine microparticles under the same treatment conditions. (**F**) Nitrite levels following exposure to SARS-CoV-2 vaccine microparticles alone or combined with Blank PLGA microparticles, GDF, Alum, or AddaVax formulations. Nitrite concentrations were quantified using the Griess assay, and data are presented as mean ± SEM (*n* = 6). Statistical significance was determined using one-way ANOVA followed by Tukey’s multiple-comparisons test. ns, *p* > 0.05; * *p* < 0.05; ** *p* < 0.01; *** *p* < 0.001; **** *p* < 0.0001.

Nitric oxide production by dendritic cells was used as a functional sign of innate immune activation to evaluate the immunostimulatory potential of GDF-loaded microparticles (GDF MPs). Nitrite accumulation in DC2.4 cell culture supernatants was quantified using the Griess assay following exposure to antigen-containing microparticle formulations administered alone or in combination (Figure 3A–F).

As shown in Figure 3A, dendritic cells treated with GDF MPs exhibited a significantly elevated nitric oxide response compared with untreated controls, confirming the immunostimulatory capacity of the formulation. Blank PLGA microparticles were included as carrier controls to evaluate the contribution of the PLGA delivery system to nitric oxide production. Nitrite levels induced by GDF MPs were similar to those generated by Alum and AddaVax microparticles, indicating a similar magnitude of innate immune activation across adjuvant systems.

Nitric oxide responses associated with Influenza H3N2 vaccine microparticles are shown in Figure 3B. Dendritic cells exposed to H3N2 microparticles in combination with GDF MPs demonstrated increased nitrite production compared with cells treated with H3N2 microparticles alone. Nitric oxide levels were comparable across formulations containing GDF MPs, Alum, or AddaVax, with all adjuvant groups exhibiting elevated responses relative to vaccine-only controls. Nitric oxide production following exposure to gonorrhea vaccine microparticles is presented in Figure 3C. Co-delivery of GDF MPs with gonorrhea vaccine microparticles resulted in a significant increase in nitrite release relative to the vaccine microparticles alone. Nitric oxide levels across adjuvant groups (GDF, Alum, and AddaVax) were of similar magnitude, although minor variations were observed between formulations. Formulations containing GDF MPs together with Alum or AddaVax exhibited elevated nitric oxide responses relative to single-adjuvant formulations, suggesting enhanced nitric oxide production in combination adjuvant formulations. For the Zika vaccine (Figure 3D), co-administration with GDF MPs significantly enhanced nitric oxide production compared with vaccine-only formulations. Nitrite levels observed with GDF formulations were similar to those obtained with Alum- and AddaVax-based systems. Combination formulations containing GDF MPs with Alum or AddaVax resulted in increased nitric oxide production relative to GDF MPs alone. As illustrated in Figure 3E, dendritic cells treated with measles vaccine microparticles combined with GDF MPs exhibited significantly elevated nitric oxide production compared with measles vaccine microparticles alone. The response induced by the GDF formulation was comparable to or higher than that observed with Alum and similar to AddaVax. Inclusion of Alum or AddaVax alongside GDF MPs further modulated nitric oxide production, indicating antigen- and formulation-dependent effects on innate immune activation.

Similarly, nitric oxide release induced by SARS-CoV-2 vaccine microparticles is shown in Figure 3F. Combination treatment with GDF MPs led to a significant increase in nitrite production relative to SARS-CoV-2 vaccine microparticles alone. The GDF formulation produced nitric oxide levels comparable to or higher than those observed with Alum and similar to AddaVax. Combination formulations containing GDF MPs with Alum or AddaVax further modulated nitric oxide output.

### 3.3. Evaluation of Guanine-α-D-Fructose Release from Polymeric Microparticles Under In Vitro Conditions

**Figure 4:**
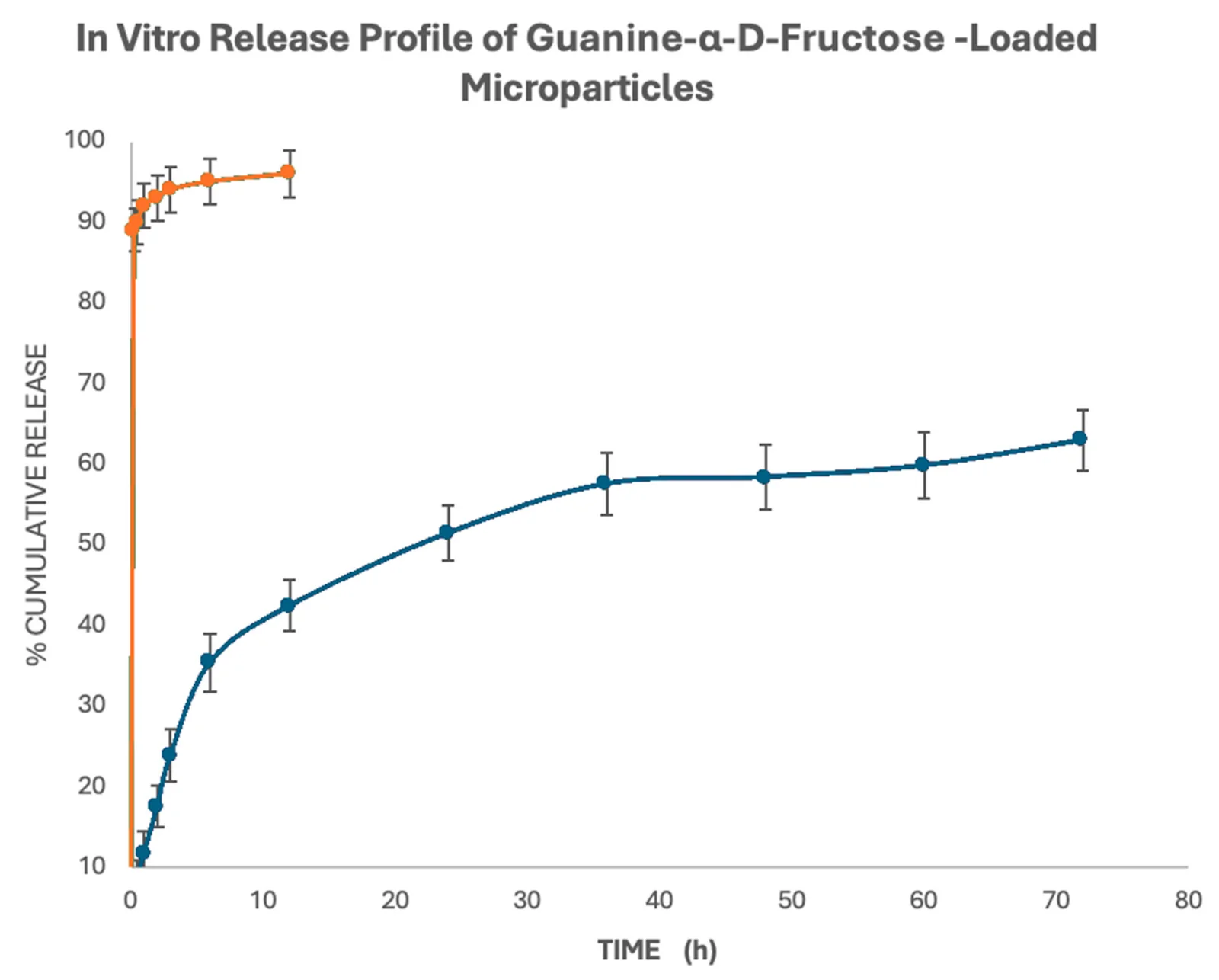
In vitro cumulative release profile of Guanine-α-D-Fructose from PLGA microparticles and free GDF. The orange curve represents free GDF, while the blue curve represents GDF-loaded PLGA microparticles. The release kinetics of GDF from the microparticle formulation were evaluated in phosphate-buffered saline (PBS, pH 7.4) at 37 °C over 72 h. The microparticle formulation exhibited a sustained release profile, with approximately 50% of the encapsulated compound released after ~20 h. Free GDF exhibited rapid release, with approximately 90% released within 15 min and ~94–95% released shortly thereafter. At designated sampling intervals, aliquots were withdrawn and replaced with an equal volume of fresh buffer to maintain sink conditions throughout the experiment. Data are presented as mean ± SEM (*n* = 5).

The in vitro release profile of GDF from PLGA microparticles was monitored over a 72 h period to characterize release dynamics. As shown in Figure 4, the cumulative release curve evidenced a biphasic arrangement, showing an initial rapid increase in GDF release during the early time points, succeeded by a slower, sustained release phase extending through the remainder of the study. The early phase was characterized by a steep rise in cumulative release within the first several hours, whereas subsequent time points showed a gradual increase approaching a plateau by 72 h, indicating controlled release from the polymeric matrix.

In contrast, free GDF displayed a rapid burst release profile, with approximately 90% of the compound released within the first 15 min and reaching near-complete release shortly thereafter. Because both free GDF and GDF-loaded microparticles were evaluated using the same dialysis bag setup, the rapid appearance of free GDF in the external medium served as a diffusion control, whereas the delayed appearance of GDF from microparticles reflected controlled release from the PLGA matrix. This stark difference highlights the ability of PLGA microparticles to effectively modulate release kinetics, minimizing the burst effect and enabling sustained delivery over extended time periods.

**Figure 5:**
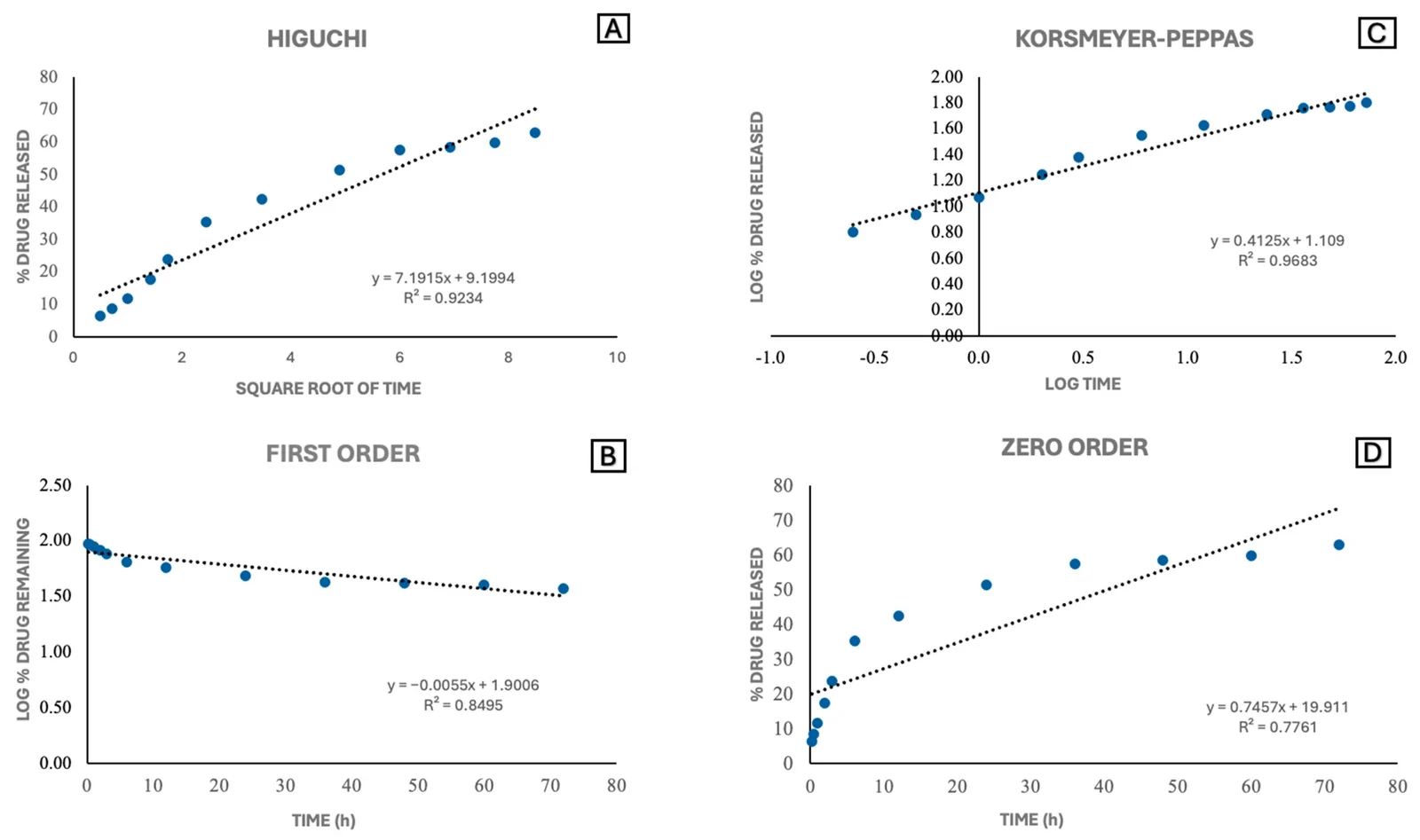
Mathematical modeling of release kinetics for the microparticle formulation. The experimental release data were subjected to multiple kinetic models to evaluate the mechanism of compound release. Panel (**A**) corresponds to the Higuchi diffusion model (R^2^ = 0.9234), while panel (**B**) shows the first-order kinetic model (R^2^ = 0.8495). Panel (**C**) illustrates the Korsmeyer–Peppas model with an R^2^ value of 0.9683, and panel (**D**) represents the zero-order model (R^2^ = 0.7761). The data points represent the experimental release data, while the dashed lines represent the corresponding linear regression fits for each kinetic model.

To further elucidate the release mechanism, the experimental data points were fitted to several commonly used kinetic models (Table 3). Fitting the data to the Higuchi model (Figure 5A) demonstrated a comparatively high correlation coefficient (R^2^ = 0.9234), consistent with diffusion-controlled release from the polymeric matrix. Analysis using the first-order model (Figure 5B) resulted in a comparatively lower correlation coefficient (R^2^ = 0.8495), indicating reduced agreement with the observed release profile relative to the Higuchi and Korsmeyer–Peppas models. Additional evaluation using the Korsmeyer–Peppas model (Figure 5C) demonstrated the highest correlation coefficient (R^2^ = 0.9683) across the evaluated models, suggesting strong agreement between the model and the observed release profile. The release exponent (*n*), obtained from the slope of the Korsmeyer–Peppas plot equation (y = 0.4125x + 1.109), was 0.4125, which is indicative of Fickian diffusion-controlled release. Linear regression using the zero-order model (Figure 5D) demonstrated the lowest correlation coefficient (R^2^ = 0.7761), suggesting that release was not governed by a constant-rate mechanism. Collectively, these findings indicate that GDF-loaded PLGA microparticles exhibit a sustained release profile dominated primarily by diffusion-mediated release behavior.

### 3.4. Evaluation of Cytocompatibility of Guanine-α-D-Fructose (GDF) Microparticles

The cytocompatibility of GDF-loaded microparticles was examined using dendritic cells as a representative antigen-presenting cell model. Cell viability was assessed using the MTT assay to determine potential cytotoxic effects following in vitro exposure. Untreated cells served as the negative control to establish baseline viability, while cells exposed to dimethyl sulfoxide (DMSO) were used as a positive control to confirm assay responsiveness.

**Figure 6:**
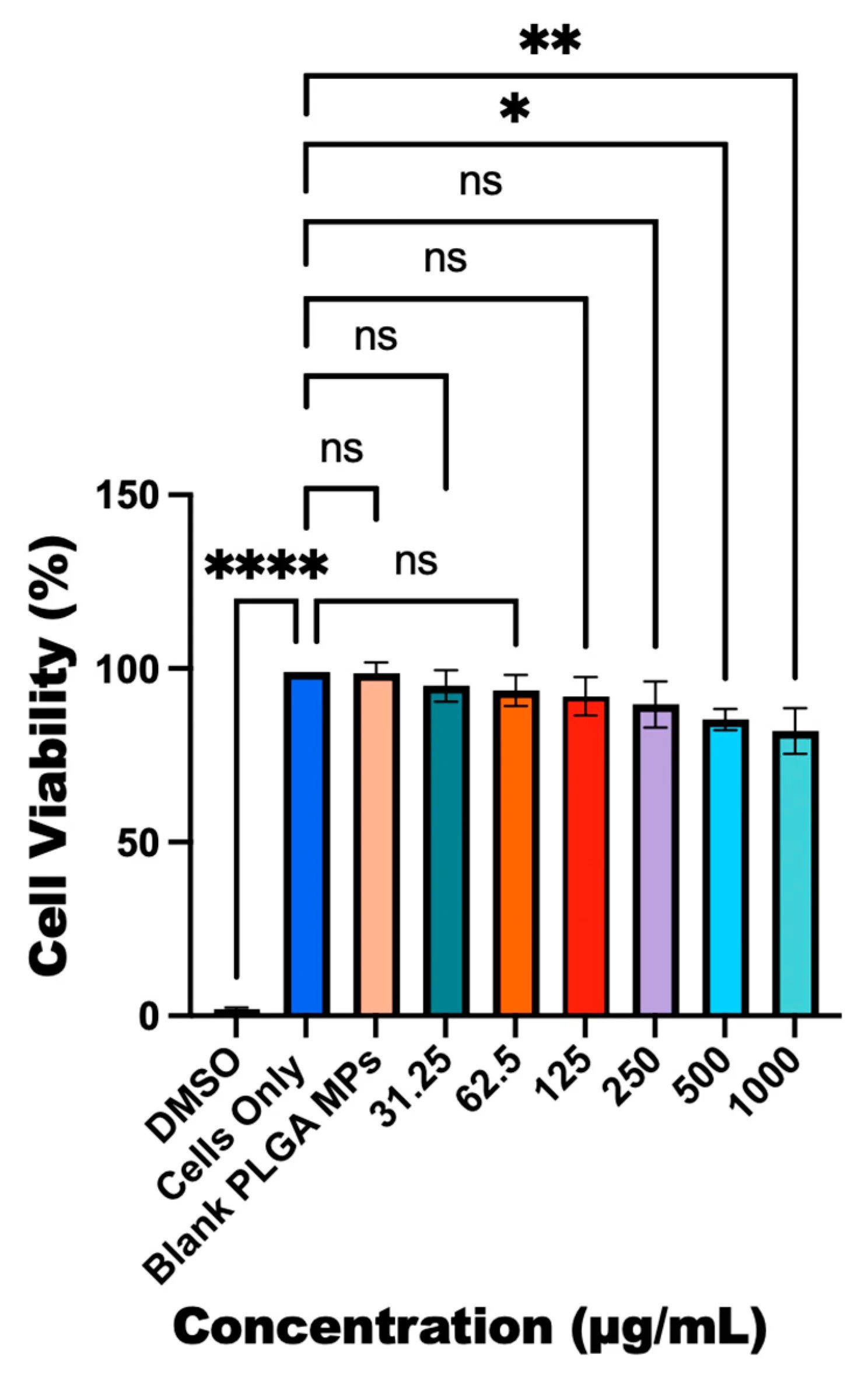
Effect of Guanine-α-D-Fructose microparticles (GDF MPs) on dendritic cells. DC2.4 murine dendritic cells were cultured for 24 h after being subjected to progressively higher doses of GDF MPs. MTT (3-(4,5-dimethylthiazol-2-yl)-2,5-diphenyl tetrazolium bromide) reagent, which uses the reducing capacity of living cells to quantitatively quantify cell viability, was used to analyze cytotoxicity. DMSO was used as a positive control for cytotoxicity. Data are presented as mean ± SEM (*n* = 6). Statistical significance was determined using one-way ANOVA followed by Tukey’s multiple-comparisons test. ns, *p* > 0.05; * *p* < 0.05; ** *p* < 0.01; **** *p* < 0.0001.

Dendritic cells were treated with a range of GDF microparticle concentrations to assess dose-dependent effects on cell viability. Across the tested concentration range, GDF microparticles did not induce a statistically significant reduction in cell viability at concentrations up to 250 μg/mL. However, at higher concentrations (>250 μg/mL), a statistically significant decrease in viability was observed, indicating dose-dependent cytotoxic effects at elevated doses. These findings indicate that GDF microparticles exhibit favorable cytocompatibility at lower concentrations, with reduced viability observed only at higher doses (Figure 6).

### 3.5. Flow Cytometric Analysis of Antigen-Presenting and Co-Stimulatory Marker Expression

To define how the GDF microparticle system modulates dendritic cell activation in a vaccine context, surface levels of MHC I, MHC II, CD80, and CD40 were quantified by flow cytometry following exposure to vaccine microparticles alone or in combination with established adjuvants (Alum MPs, AddaVax MPs) and GDF-containing formulations.

**Figure 7:**
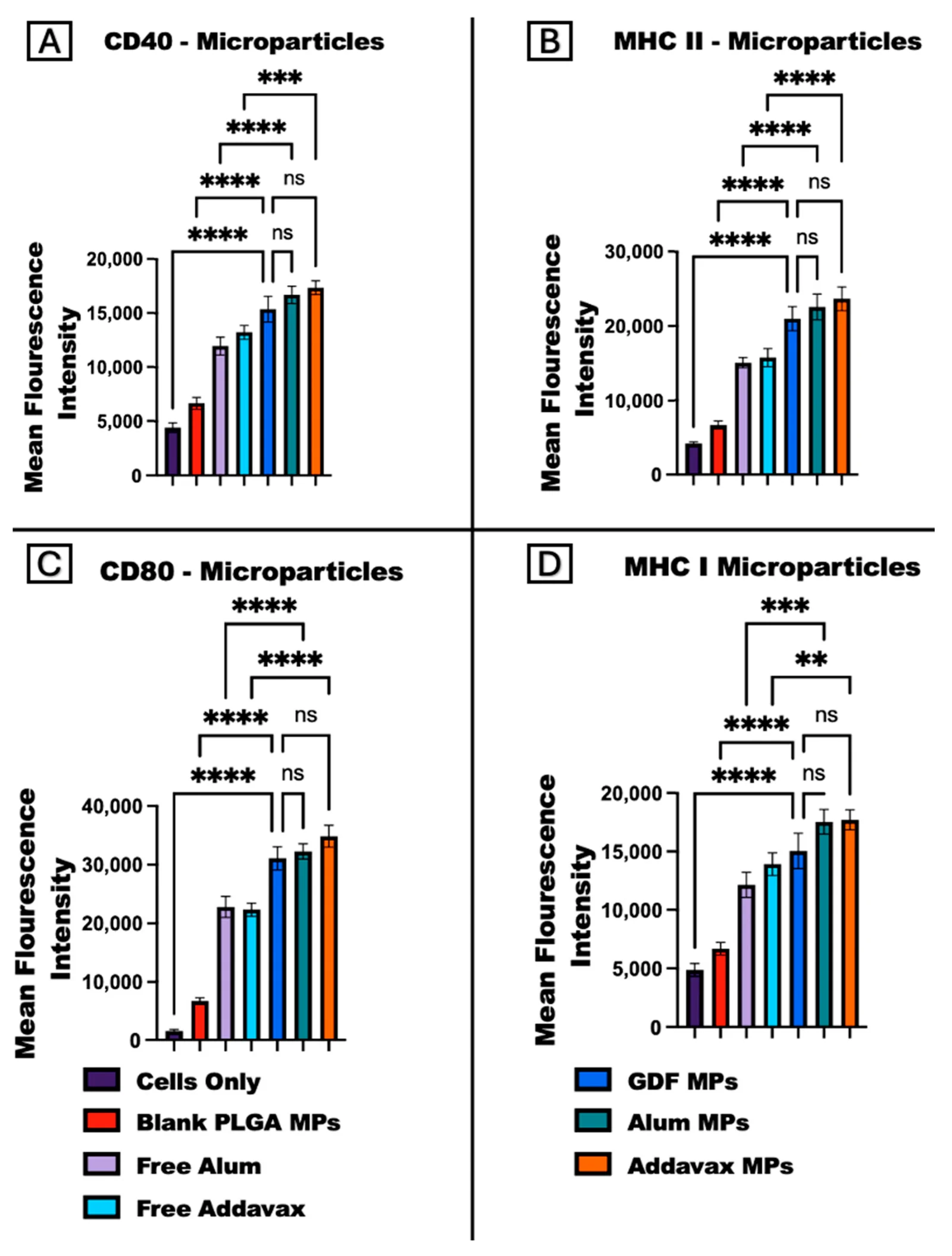
Flow cytometric evaluation of dendritic cell surface marker expression following treatment with Blank PLGA microparticles, free Alum, free AddaVax, Guanine-α-D-Fructose microparticles, Alum microparticles, and AddaVax microparticles. (**A**) CD40 expression, (**B**) MHC II expression, (**C**) CD80 expression, and (**D**) MHC I expression in murine dendritic cells. Untreated cells served as the negative control. Blank PLGA microparticles were included as carrier controls to evaluate the contribution of the PLGA delivery system to dendritic cell marker expression. Marker expression is presented as mean fluorescence intensity ± SEM (*n* = 6). Statistical significance was determined using one-way ANOVA followed by Tukey’s multiple-comparisons test. ns, *p* > 0.05; ** *p* < 0.01; *** *p* < 0.001; **** *p* < 0.0001.

Flow cytometric evaluation revealed that microparticle-based adjuvant formulations markedly enhanced dendritic cell activation relative to untreated controls. As shown in Figure 7, exposure to GDF microparticles resulted in a pronounced increase in surface expression of both antigen-presenting molecules (MHC I and MHC II) and co-stimulatory markers (CD40 and CD80) compared with cells alone. For all four markers examined, the untreated group exhibited minimal baseline expression, whereas adjuvanted formulations produced substantially elevated mean fluorescence intensities.

When compared against benchmark adjuvants, GDF microparticles demonstrated activation profiles comparable to Alum and AddaVax across all markers examined. All adjuvant-treated groups showed significantly increased expression relative to untreated controls, with no statistically significant differences observed among GDF-, Alum-, and AddaVax-containing formulations. Together, these findings indicate that GDF microparticles induce robust dendritic cell activation across both antigen-presentation and co-stimulatory pathways.

**Figure 8:**
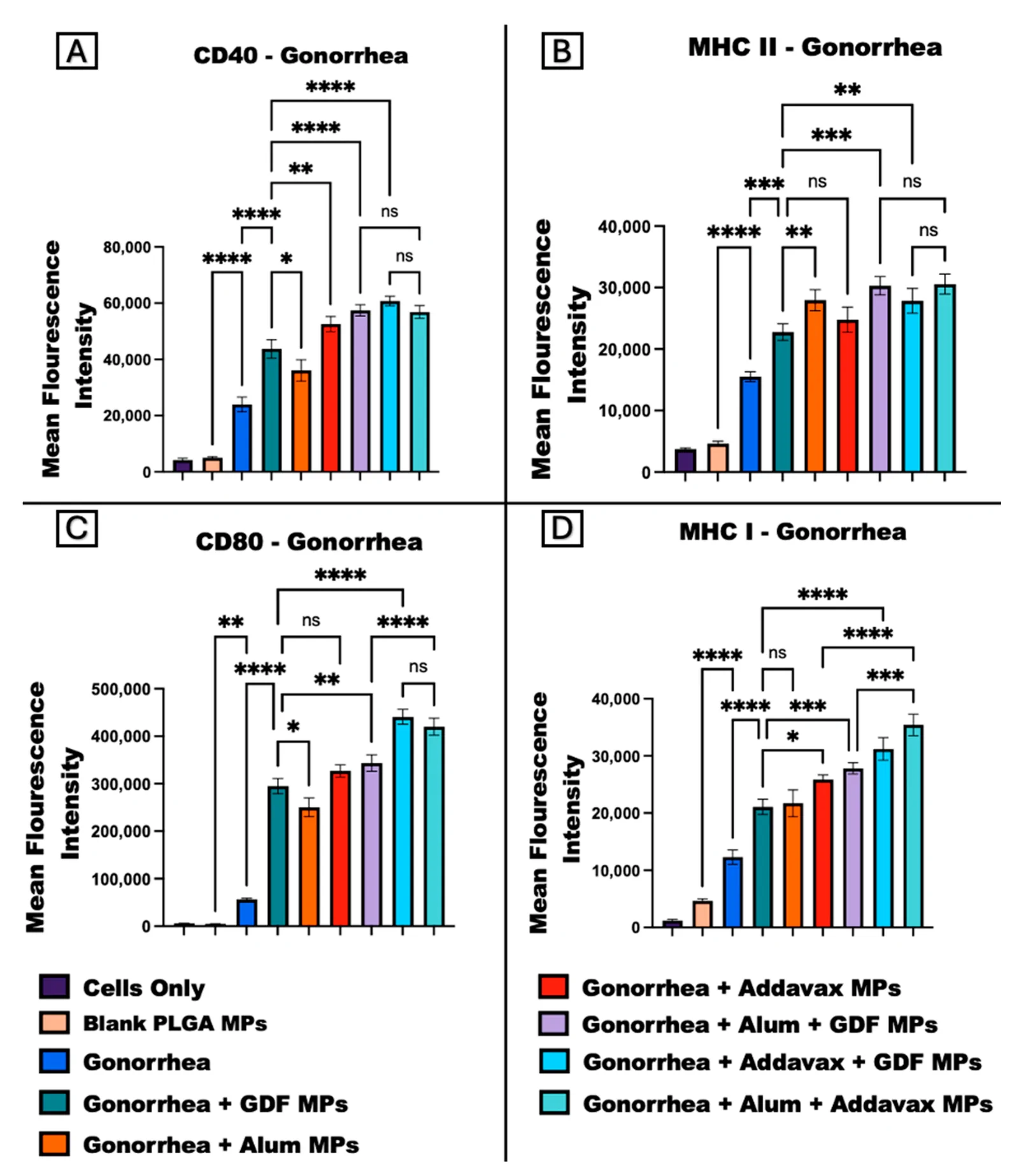
Dendritic cell activation following exposure to gonorrhea vaccine formulations containing Guanine-α-D-Fructose microparticles. (**A**) Surface expression of CD40 on murine dendritic cells following treatment with gonorrhea vaccine microparticles alone or in combination with GDF microparticles, Alum microparticles, AddaVax microparticles, or their respective combinations. (**B**) Surface expression of MHC II on murine dendritic cells following treatment with gonorrhea vaccine microparticles under the same conditions. (**C**) Surface expression of CD80 on murine dendritic cells following treatment with gonorrhea vaccine microparticles under the same conditions. (**D**) Surface expression of MHC I on murine dendritic cells following treatment with gonorrhea vaccine microparticles under the same conditions. Blank PLGA microparticles were included as carrier controls. Data are presented as mean ± SEM (*n* = 6). Statistical significance was determined using one-way ANOVA followed by Tukey’s multiple-comparisons test. ns, *p* > 0.05; * *p* < 0.05; ** *p* < 0.01; *** *p* < 0.001; **** *p* < 0.0001.

To evaluate the immunostimulatory contribution of GDF microparticles in the context of a gonorrhea vaccine, dendritic cells were analyzed for surface expression of antigen-presentation and co-stimulatory markers following exposure to vaccine microparticles alone or in combination with adjuvant formulations (Figure 8). Across all markers examined, vaccine-only treatment resulted in a marked increase relative to untreated cells; however, incorporation of adjuvant microparticles further amplified dendritic cell activation.

Overall, GDF-containing gonorrhea vaccine formulations produced stronger dendritic cell activation than vaccine alone, with increased expression across both antigen-presentation and co-stimulatory markers. Responses were generally comparable to AddaVax and, in some cases, exceeded those observed with Alum. Combination formulations further enhanced activation for selected markers, suggesting additive immunostimulatory effects.

**Figure 9:**
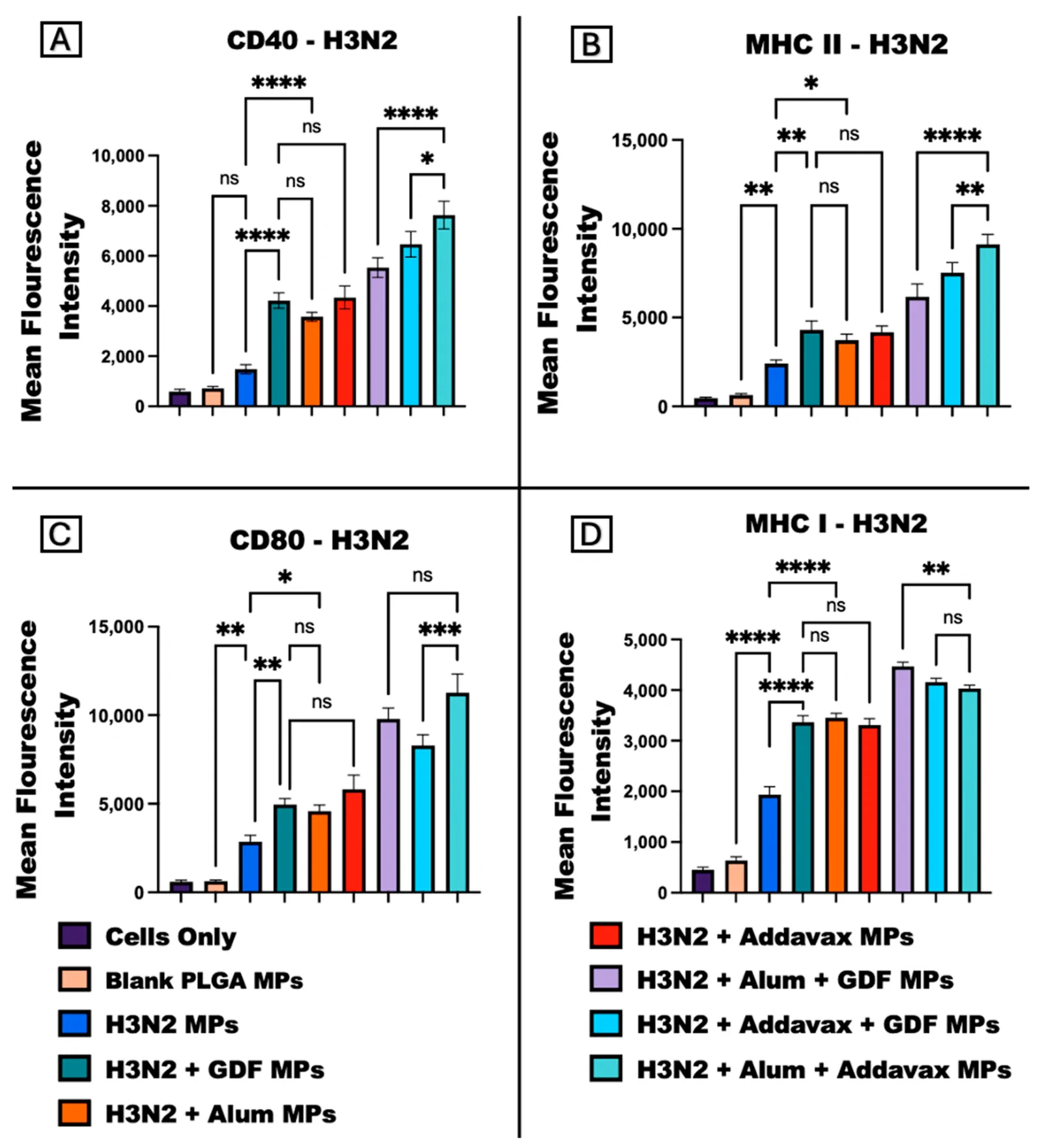
Dendritic cell activation induced by H3N2 vaccine formulations containing Guanine-α-D-Fructose microparticles. (**A**) Surface expression of CD40 on murine dendritic cells following exposure to H3N2 vaccine microparticles alone or in combination with GDF microparticles, Alum microparticles, AddaVax microparticles, or their respective combinations. (**B**) Surface expression of MHC II on murine dendritic cells following exposure to H3N2 vaccine microparticles under the same conditions. (**C**) Surface expression of CD80 on murine dendritic cells following exposure to H3N2 vaccine microparticles under the same conditions. (**D**) Surface expression of MHC I on murine dendritic cells following exposure to H3N2 vaccine microparticles under the same conditions. Blank PLGA microparticles were included as carrier controls. Data are presented as mean ± SEM (*n* = 6). Statistical significance was determined using one-way ANOVA followed by Tukey’s multiple-comparisons test. ns, *p* > 0.05; * *p* < 0.05; ** *p* < 0.01; *** *p* < 0.001; **** *p* < 0.0001.

The immunostimulatory effect of GDF microparticles in the context of an H3N2 vaccine was examined by quantifying dendritic cell surface expression of antigen-presenting and co-stimulatory markers (Figure 9). Treatment with H3N2 vaccine alone increased marker expression relative to untreated cells; however, incorporation of adjuvant microparticles produced a more pronounced activation profile across all markers evaluated.

Overall, inclusion of GDF microparticles enhanced dendritic cell activation relative to H3N2 vaccine alone. Marker expression was comparable to AddaVax and generally similar to or stronger than Alum depending on the parameter evaluated, while combination formulations produced the highest activation levels.

**Figure 10:**
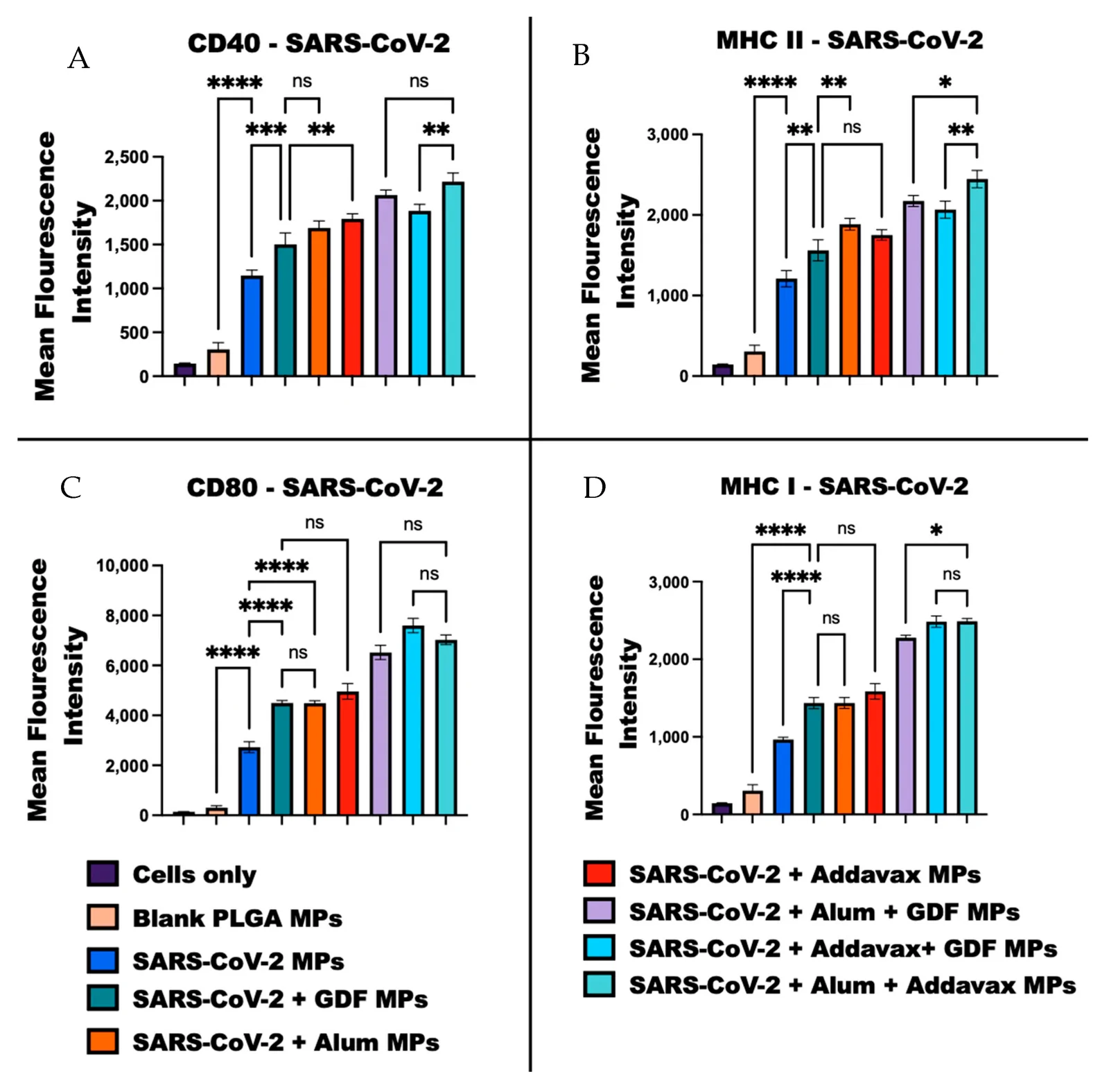
Dendritic cell activation following exposure to SARS-CoV-2 Omicron vaccine formulations containing Guanine-α-D-Fructose microparticles. (**A**) Surface expression of CD40 on murine dendritic cells following exposure to SARS-CoV-2 vaccine alone or in combination with GDF microparticles, Alum microparticles, AddaVax microparticles, or their respective combinations. (**B**) Surface expression of MHC II on murine dendritic cells following exposure to SARS-CoV-2 vaccine under the same conditions. (**C**) Surface expression of CD80 on murine dendritic cells following exposure to the SARS-CoV-2 vaccine under the same conditions. (**D**) Surface expression of MHC I on murine dendritic cells following exposure to the SARS-CoV-2 vaccine under the same conditions. Blank PLGA microparticles were included as carrier controls. Data are presented as mean ± SEM (*n* = 6). Statistical significance was determined using one-way ANOVA followed by Tukey’s multiple-comparisons test. ns, *p* > 0.05; * *p* < 0.05; ** *p* < 0.01; *** *p* < 0.001; **** *p* < 0.0001.

The immunostimulatory capacity of GDF-containing formulations in the context of the SARS-CoV-2 vaccine was evaluated by quantifying dendritic cell surface expression of co-stimulatory and antigen-presentation markers (Figure 10). Across all markers examined, vaccine-only treatment increased expression relative to untreated cells; however, incorporation of adjuvant microparticles further amplified dendritic cell activation.

GDF-containing formulations produced particularly strong increases in dendritic cell activation in the SARS-CoV-2 vaccine model, with responses comparable to AddaVax and exceeding Alum in selected comparisons. Combination formulations further amplified marker expression, supporting the ability of GDF to function effectively within multi-adjuvant systems.

**Figure 11:**
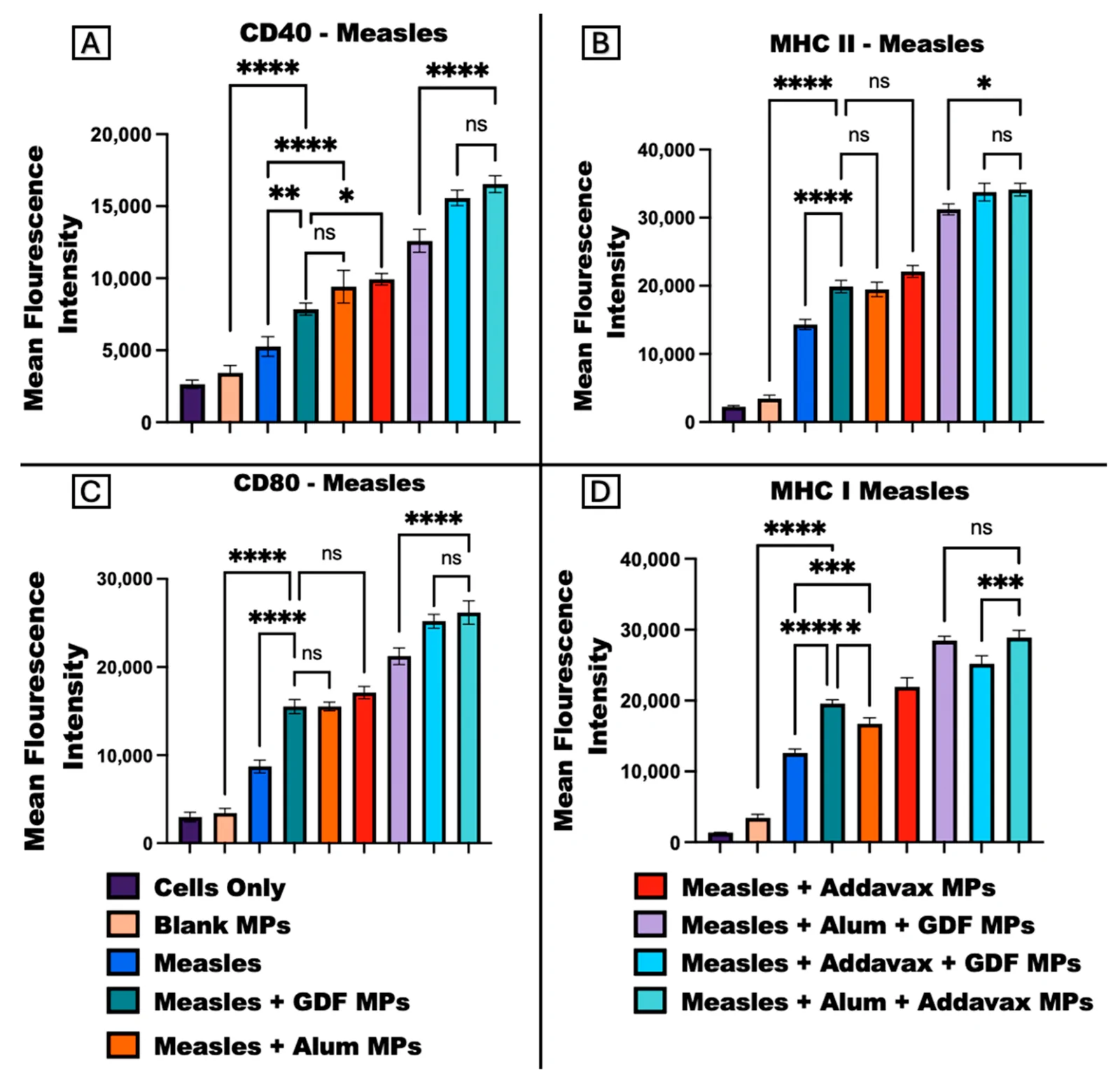
Dendritic cell activation following exposure to measles vaccine formulations containing Guanine-α-D-Fructose microparticles. (**A**) Surface expression of CD40 on murine dendritic cells following exposure to measles vaccine alone or in combination with GDF microparticles, Alum microparticles, AddaVax microparticles, or their respective combinations. (**B**) Surface expression of MHC II on murine dendritic cells following exposure to measles vaccine under the same conditions. (**C**) Surface expression of CD80 on murine dendritic cells following exposure to measles vaccine under the same conditions. (**D**) Surface expression of MHC I on murine dendritic cells following exposure to measles vaccine under the same conditions. Blank PLGA microparticles were included as carrier controls. Data are presented as mean ± SEM (*n* = 6). Statistical significance was determined using one-way ANOVA followed by Tukey’s multiple-comparisons test. ns, *p* > 0.05; * *p* < 0.05; ** *p* < 0.01; *** *p* < 0.001; **** *p* < 0.0001.

Dendritic cell activation in response to measles vaccine formulations was evaluated by measuring surface expression of co-stimulatory molecules (CD40, CD80) and antigen-presentation markers (MHC I, MHC II) following exposure to vaccine alone or adjuvanted formulations (Figure 11). Across all markers, untreated cells displayed minimal baseline expression, while measles vaccine alone induced a clear increase. Notably, incorporation of adjuvant microparticles further amplified dendritic cell activation in a formulation-dependent manner.

In the measles vaccine model, GDF microparticles significantly enhanced dendritic cell activation relative to vaccine alone, with responses similar to those observed with established adjuvants. As in the other systems, combination formulations yielded the highest overall levels of activation.

**Figure 12:**
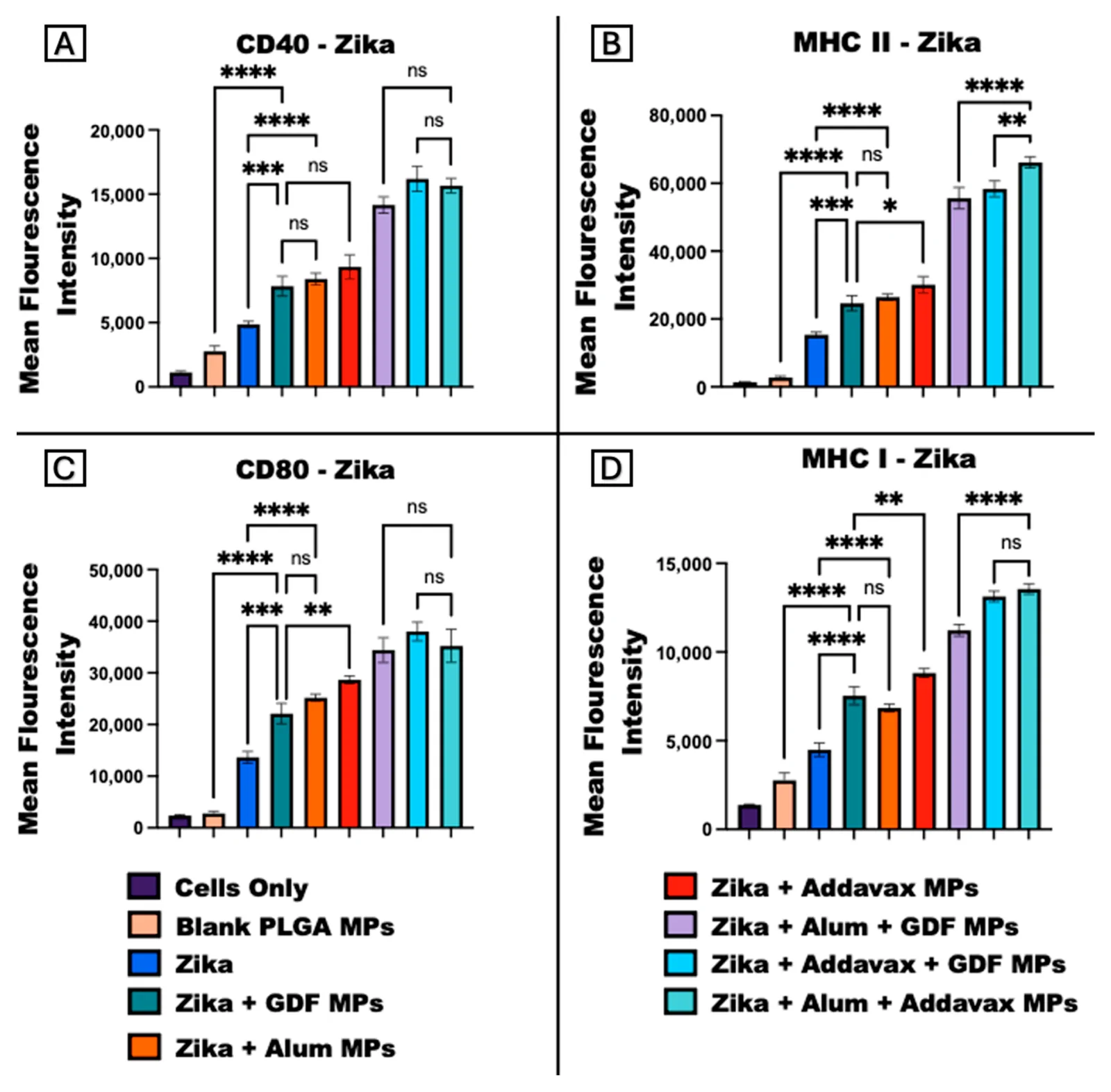
Dendritic cell activation following exposure to Zika vaccine formulations containing Guanine-α-D-Fructose microparticles. (**A**) Surface expression of CD40 on murine dendritic cells following exposure to Zika vaccine alone or in combination with GDF microparticles, Alum microparticles, AddaVax microparticles, or their respective combinations. (**B**) Surface expression of MHC II on murine dendritic cells following exposure to the Zika vaccine under the same conditions. (**C**) Surface expression of CD80 on murine dendritic cells following exposure to the Zika vaccine under the same conditions. (**D**) Surface expression of MHC I on murine dendritic cells following exposure to the Zika vaccine under the same conditions. Blank PLGA microparticles were included as carrier controls. Data are presented as mean ± SEM (*n* = 6). Statistical significance was determined using one-way ANOVA followed by Tukey’s multiple-comparisons test. ns, *p* > 0.05; * *p* < 0.05; ** *p* < 0.01; *** *p* < 0.001; **** *p* < 0.0001.

Dendritic cell activation in response to Zika vaccine formulations was evaluated by quantifying surface expression of co-stimulatory molecules (CD40, CD80) and antigen-presentation markers (MHC I, MHC II) following exposure to vaccine alone or in combination with adjuvant microparticles (Figure 12). Across all markers, untreated cells exhibited minimal baseline expression, whereas Zika vaccine alone induced a clear activation response that was further amplified by adjuvant incorporation.

Consistent with the other vaccine models, GDF-containing Zika formulations enhanced dendritic cell activation across antigen-presentation and co-stimulatory markers relative to vaccine-only controls. Activation levels were comparable to AddaVax and generally stronger than or similar to Alum, with combination formulations producing the greatest overall response.

Taken together, these findings demonstrate that GDF-loaded microparticles consistently enhance dendritic cell activation across multiple particulate vaccine formulations. GDF promoted coordinated upregulation of both antigen-presentation and co-stimulatory pathways, with activity comparable to established adjuvants and, in some cases, exceeding Alum.

### 3.6. Analysis of Autophagosome-Associated Fluorescence in Dendritic Cells

**Figure 13:**
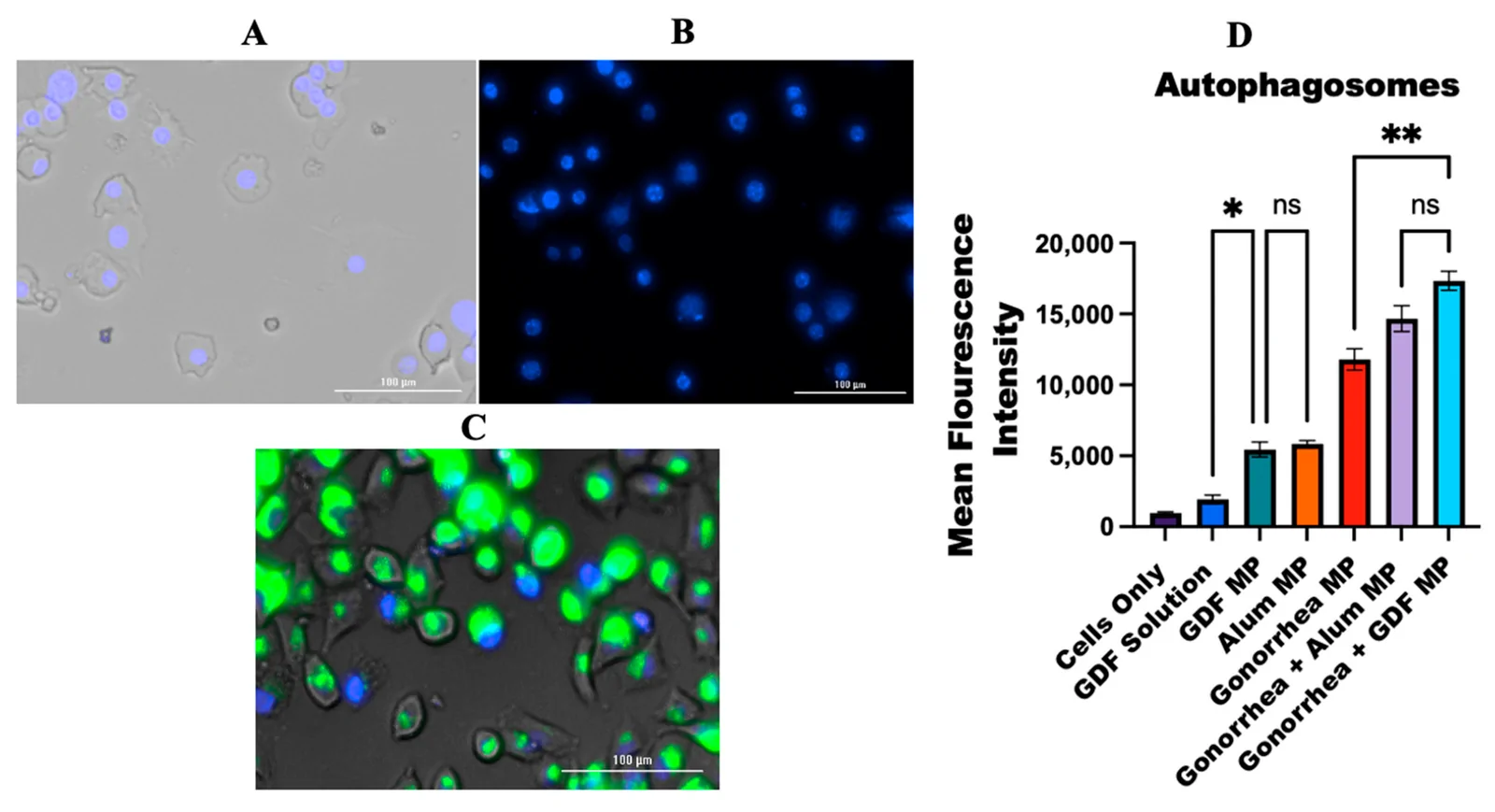
Evaluation of autophagy-associated fluorescence in DC2.4 cells. Panels (**A**–**D**) show representative fluorescence microscopy images of DC2.4 cells stained with Hoechst 33342 and CYTO-ID. Panel (**A**) represents untreated control cells, panel (**B**) cells treated with soluble Guanine-α-D-Fructose (GDF solution), and panel (**C**) cells treated with rapamycin as a positive control for autophagosome formation. Blue fluorescence represents Hoechst 33342 nuclear staining, while green fluorescence represents CYTO-ID-positive autophagic vesicles. Panel (**D**) shows quantitative flow cytometric analysis of autophagosome-associated fluorescence following treatment with GDF solution, GDF MPs, Alum microparticles, gonorrhea vaccine microparticles, and gonorrhea vaccine microparticles combined with Alum or GDF microparticles. Data are presented as mean ± SEM (*n* = 6). Statistical significance was determined using one-way ANOVA followed by Tukey’s multiple-comparisons test. ns, *p* > 0.05; * *p* < 0.05; ** *p* < 0.01.

**Figure 14:**
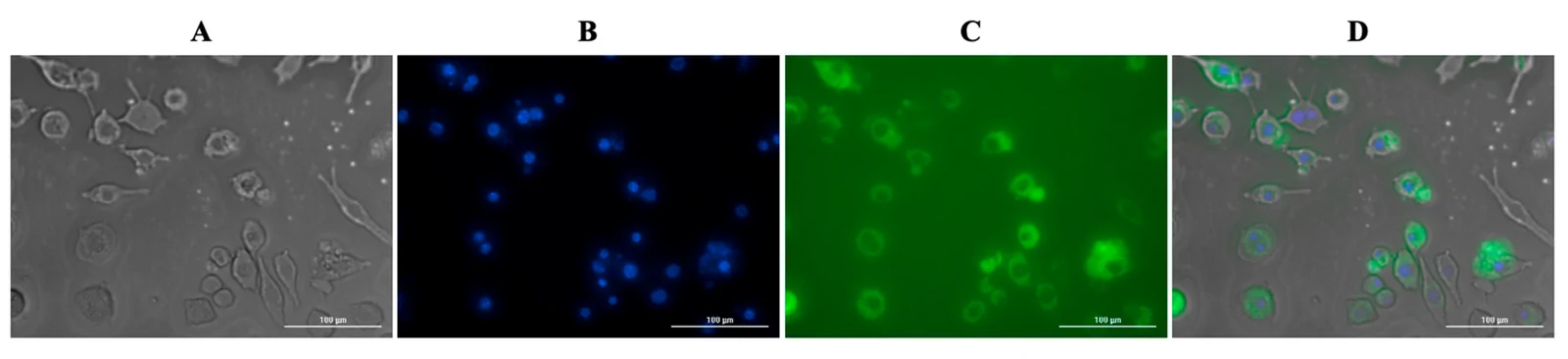
Representative fluorescence microscopy images of DC2.4 cells treated with GDF-loaded PLGA microparticles (GDF MPs). (**A**) Brightfield image, (**B**) Hoechst 33342 fluorescence showing cell nuclei in blue, (**C**) CYTO-ID fluorescence showing autophagy-associated vesicles in green, and (**D**) corresponding merged image. The separated imaging channels provide clearer visualization and interpretation of the nuclear and autophagy-associated fluorescence signals following treatment with GDF-loaded microparticles.

Autophagy-associated fluorescence in dendritic cells was evaluated using fluorescence microscopy and flow cytometry. Representative fluorescence images of untreated cells, soluble GDF-treated cells, and rapamycin-treated cells are shown in Figure 13A–C, respectively. Untreated cells demonstrated minimal autophagy-associated fluorescence, whereas cells treated with soluble GDF showed detectable intracellular CYTO-ID fluorescence. Rapamycin-treated cells, included as a positive control, exhibited strong autophagy-associated fluorescence. To provide a clearer visualization of the response following treatment with GDF-loaded microparticles, Figure 14A–D presents the GDF MP-treated cells separately as brightfield, Hoechst 33342 (blue channel), CYTO-ID (green channel), and corresponding merged images. Pronounced punctate CYTO-ID fluorescence was observed following GDF MP treatment, supporting an increase in autophagy-associated vesicle fluorescence compared with untreated cells.

Quantitative flow cytometric analysis is presented in Figure 13D and further supported the fluorescence microscopy observations. Treatment with GDF-loaded microparticles significantly increased autophagosome-associated fluorescence intensity compared with untreated and soluble GDF-treated cells. Increased fluorescence was also observed when gonorrhea vaccine microparticles were combined with either Alum or GDF microparticles. Among the evaluated formulations, the gonorrhea + GDF microparticle group demonstrated the highest autophagosome-associated fluorescence intensity; however, no statistically significant difference was observed between the gonorrhea + GDF and gonorrhea + Alum microparticle groups.

## 4. Discussion

The development of next-generation vaccine adjuvants requires the careful integration of immunostimulatory potency, cell viability, and formulation versatility. As vaccine antigens increasingly shift toward purified subunits, inactivated pathogens, and recombinant platforms, the need for adjuvant systems capable of robustly engaging innate immunity while maintaining a favorable safety profile has become increasingly apparent. In this study, we introduce and evaluate Guanine-α-D-Fructose (GDF) formulated within PLGA microparticles as an immunostimulatory particulate system and demonstrate its ability to activate dendritic cells across a diverse panel of bacterial and viral vaccine formulations. The collective findings establish GDF-loaded PLGA microparticles as a promising immunostimulatory system [2,6].

A foundational requirement for any particulate immunostimulatory delivery system is the generation of reproducible and structurally well-defined particles. Physicochemical characterization of GDF-loaded PLGA microparticles demonstrated a reproducible low-micron formulation, with a mean hydrodynamic diameter of 1.436 ± 0.049 µm, a polydispersity index of 0.21, high recovery yield, efficient GDF entrapment, and a moderately negative surface charge. These formulation attributes are important because particle size, size distribution, and surface properties can influence colloidal behavior, cellular interaction, and biological performance of polymeric particulate delivery systems [36,37]. Particle size characterization was based on hydrodynamic diameter and distribution-based particle size measurements obtained from DLS analysis. These physicochemical characteristics are relevant for interaction with antigen-presenting cells, including dendritic cells and macrophages, through phagocytic and endocytic mechanisms. The approximately 1.4 µm hydrodynamic diameter observed in the present study places the formulation within a low-micron particulate range rather than a nanoparticle range optimized for passive lymphatic trafficking. PLGA particle-size studies have shown that particle diameter can influence dendritic cell internalization, MHC I-associated antigen presentation, and antigen-specific CD8^+^ T-cell responses. Although smaller nano- and submicron-sized particles may enhance lymphatic trafficking and cross-presentation efficiency, low-micron PLGA microparticles remain relevant for vaccine delivery because they can support antigen-presenting cell interaction and sustained antigen or adjuvant availability [9,10]. The observed negative zeta potential likely contributes to colloidal stability in aqueous environments while avoiding excessive nonspecific membrane disruption [38]. SEM imaging further confirmed the spherical morphology and surface integrity of the microparticles, indicating that the encapsulation process preserved structural robustness [39]. Together, these properties provided a stable and biologically suitable platform for immunological evaluation. In this context, the approximately 1.4 µm size, moderate polydispersity, spherical morphology, and sustained release behavior of the GDF-loaded PLGA microparticles provided an appropriate platform for evaluating dendritic cell activation in vitro, while direct assessment of humoral and antigen-specific T-cell responses will require future in vivo studies. The estimated entrapment efficiency of GDF-loaded PLGA microparticles indicated efficient incorporation of GDF within the polymeric carrier system. This is important for interpretation of biological dosing, as the administered microparticles contain a substantial retained GDF fraction within the particulate matrix.

Previous investigations from our group have demonstrated that PLGA-based vaccine microparticles fabricated using double-emulsion solvent evaporation techniques can achieve high levels of antigen incorporation efficiency. Using comparable formulation strategies, particle size and encapsulation efficiencies of approximately 1413 ± 117 nm and 92% were previously reported for H3N2 microparticles [16], 731 ± 21 nm and 89.1% for SARS-CoV-2 Omicron variant antigen-loaded microparticles [24], 533 ± 10 nm with 55–70% for Zika vaccine microparticle systems and gonorrhea with 3.5 ± 1.2 µm [40]. The physicochemical behavior observed in the current study is consistent with the established formulation performance of these PLGA particulate delivery platforms. Such characteristics are important for supporting formulation stability, sustained release behavior, and reproducible interaction with antigen-presenting cells.

Innate immune activation was first functionally assessed through nitric oxide production, a well-established marker of dendritic cell activation and inflammatory signaling [41]. Across multiple particulate vaccine formulations, which include H3N2, Zika, measles, gonorrhea, and SARS-CoV-2, GDF microparticles consistently induced nitric oxide responses that were comparable to or exceeded those elicited by benchmark adjuvants such as Alum and AddaVax. GDF microparticles increased nitric oxide production when combined with antigen microparticles relative to antigen alone, supporting increased in vitro innate activation-associated responses in antigen-containing formulations rather than establishing in vivo adjuvant activity [42]. The observation that GDF-induced nitric oxide levels varied depending on antigen context suggests that the platform supports antigen-dependent tuning of innate immune responses, a desirable feature for vaccine optimization.

The release profile of GDF-loaded PLGA microparticles showed an initial faster release phase followed by slower, sustained release over 72 h, whereas free GDF diffused rapidly through the dialysis membrane within minutes. Because both samples were evaluated using dialysis bags with a molecular-weight cutoff of 3.5 kDa, the free-GDF profile reflects membrane diffusion, while the microparticle profile reflects release from the PLGA matrix followed by diffusion through the membrane. This comparison indicates that PLGA encapsulation effectively modulated GDF release under the in vitro conditions evaluated. The early release phase is likely attributable to diffusion of more readily accessible GDF located near or at the particle surface, while the later sustained phase is consistent with continued diffusion of encapsulated GDF from within the PLGA microparticle matrix. Kinetic analysis using the Korsmeyer–Peppas model provided the best fit to the release data (R^2^ = 0.9683), and the calculated release exponent (*n* = 0.4125) was below the 0.43 threshold described by Ritger and Peppas for spherical systems [43]. This supports a predominantly Fickian diffusion-controlled release mechanism under the conditions evaluated in this study. Under Fickian diffusion, drug transport is driven purely by a concentration gradient, with the rate of molecular diffusion exceeding that of polymer chain relaxation. Accordingly, GDF release is governed by passive diffusion through the polymer network rather than by matrix erosion or swelling, which is consistent with the expected behavior of PLGA microparticles during the early-to-intermediate stages of release.

Overall, these findings indicate that PLGA microparticle encapsulation converted the rapid release behavior of free GDF into a sustained, diffusion-controlled release profile, which may be beneficial for maintaining longer GDF availability during dendritic cell exposure [44,45].

Equally critical to formulation development is the demonstration of cytocompatibility. GDF-loaded microparticles maintained viability up to 250 µg/mL, while higher concentrations reduced viability, defining a practical concentration window for functional assays. This finding is particularly important given that several highly active immunostimulatory compounds fail to progress due to dose-limiting toxicity [36]. The absence of cytotoxic effects supports the notion that the guanine–fructose molecular design, combined with PLGA encapsulation, confers a favorable biological interface.

The ability of GDF microparticles to remain compatible within combination particulate formulations was particularly notable. In several antigen systems, co-formulation of GDF with Alum or AddaVax further increased nitric oxide production, indicating additive-like effects [46]. These findings suggest that GDF may be suitable for further evaluation in combination particulate systems alongside established adjuvants.

While nitric oxide production provides an early indicator of innate activation, effective vaccine adjuvants must ultimately promote dendritic cell activation and antigen presentation [47]. Flow cytometric analysis across all vaccine models revealed that GDF microparticles upregulated MHC class I, MHC class II, CD40, and CD80, confirming their capacity to drive dendritic cell activation at multiple functional levels. Importantly, this activation was consistently observed across both bacterial and viral antigens [46,47]. Increased expression of MHC class I and MHC class II molecules following treatment with GDF-loaded microparticles is indicative of dendritic cell activation and may reflect enhanced antigen-processing potential. Additional mechanistic studies evaluating antigen presentation pathways and antigen-specific T-cell responses would help further characterize the immunological mechanisms associated with GDF-mediated dendritic cell activation. This feature is particularly relevant for viral vaccines and intracellular pathogens, where cellular immunity plays a dominant protective role. The ability of GDF microparticles to induce MHC I expression at levels comparable to or exceeding those of AddaVax highlights their potential utility in vaccine strategies targeting viruses such as influenza, Zika, and emerging zoonotic pathogens [48,49]. Increased MHC class II surface expression is consistent with a more activated antigen-presenting-cell phenotype, but antigen processing and helper T-cell responses require direct functional confirmation. The simultaneous increase in MHC I, MHC II, CD40, and CD80 across the tested antigen formulations indicates a broad activation-associated phenotype in DC2.4 cells, involving markers linked to both MHC I- and MHC II-mediated antigen presentation as well as co-stimulatory signaling [50,51].

Under the conditions tested, GDF-loaded microparticles produced measurable immunostimulatory responses in DC2.4 cells. Some responses were similar in magnitude to those observed with Alum- and AddaVax-containing formulations. Across most antigen systems, GDF induced dendritic cell activation profiles that were statistically indistinguishable from Alum and AddaVax, and in several cases exceeded Alum-mediated responses. Moreover, the observation that GDF-containing formulations often achieved activation levels comparable to AddaVax highlights the strength of this molecule-polymer combination.

Beyond conventional activation markers, the evaluation of autophagic responses provides insight into how GDF microparticles may potentially enhance antigen presentation. Autophagy is increasingly recognized as a critical regulator of antigen processing, influencing both MHC class II presentation and MHC class I cross-presentation. The observed induction of autophagosomes in dendritic cells treated with microparticle formulations suggests that GDF may engage intracellular pathways that facilitate antigen routing and processing. This is particularly relevant given prior reports linking autophagy to improved vaccine efficacy and durable T-cell responses [52,53].

The molecular design of GDF likely contributes directly to these effects. Guanine-containing motifs have been implicated in immune recognition pathways, including interactions with pattern-recognition receptors and nucleic acid–sensing mechanisms. While the precise receptors engaged by GDF remain to be elucidated, the consistent induction of dendritic cell activation markers suggests engagement of conserved innate signaling pathways. The inclusion of a fructose moiety may further enhance cellular uptake or intracellular trafficking, potentially contributing to the observed biological activity. Encapsulation within PLGA microparticles likely amplifies these effects by promoting efficient cellular internalization, sustained release, and localized intracellular exposure [13,54].

The additive immunostimulatory activity observed with GDF-containing formulations may potentially involve innate immune signaling pathways associated with nucleobase-derived immunomodulators. Previous studies have demonstrated that certain guanine-containing nucleoside analogs can stimulate antigen-presenting cells through Toll-like receptor-mediated pathways, including TLR7-associated immune activation. In addition, water-soluble nucleobase-derived compounds such as resiquimod hydrochloride have been reported to enhance innate immune activation through related mechanisms. Although the precise molecular signaling pathways associated with GDF were not directly investigated in the present study, the observed enhancement of nitric oxide production, dendritic cell activation, and autophagosome-associated responses across multiple vaccine systems suggests that the guanine-containing structure of GDF may contribute to its immunostimulatory activity through related innate immune mechanisms. However, further mechanistic studies will be required to determine whether TLR7/8-associated signaling or alternative nucleobase-responsive pathways are directly involved in the biological activity of GDF [14,55].

Across the particulate vaccine formulations tested, GDF-loaded microparticles produced measurable immunostimulatory responses in DC2.4 cells. Although these findings do not establish broad adjuvant efficacy, the similar pattern of activity observed across several antigen-containing formulations suggests that the response was not limited to a single experimental context. These preliminary in vitro findings support further evaluation of GDF-loaded microparticles in primary immune-cell systems and in vivo vaccination models [56,57].

While the present study demonstrates clear in vitro immunostimulatory effects of GDF-loaded PLGA microparticles, additional work is needed to further define their translational potential. The GDF used in this study was obtained commercially and used as supplied for the preparation of the microparticles. No additional independent assessment of compound purity was performed as part of the present investigation, which was focused on formulation characterization and in vitro evaluation. The current findings are based on a murine dendritic cell model and therefore do not fully capture the complexity of immune responses in vivo. Future studies should evaluate antigen-specific immune responses, protective efficacy, antibody generation, T-cell polarization, persistence of immune memory, and further validate the biological activity of GDF-loaded PLGA microparticles using primary human dendritic cells and appropriate in vivo mouse vaccination models. Although nitric oxide production and increased surface marker expression indicate strong innate immune activation, a more detailed evaluation of cytokine signaling and downstream adaptive responses would help clarify the broader immunological impact of this system. Although a reduction in viability was observed at higher GDF concentrations (>250 μg/mL), the principal immunological analyses were interpreted primarily within concentration ranges that retained substantial cellular viability. Therefore, the observed increases in dendritic cell activation markers and nitric oxide production were not considered to be solely attributable to nonspecific cytotoxic stress effects. At the same time, these findings provide a strong basis for continued development. The combination of a guanine-derived small molecule with a biodegradable PLGA delivery platform offers a practical and effective approach to enhancing immune activation while maintaining cytocompatibility and formulation stability. The consistent performance observed across multiple particulate vaccine formulations, along with compatibility with established adjuvants, suggests that this system could be adapted for a range of applications and supports its progression into further preclinical studies.

## 5. Conclusions

This study provides a formulation-level basis for the further development of Guanine-α-D-Fructose (GDF)-loaded PLGA microparticles as an in vitro immunostimulatory system. The formulation exhibited defined physicochemical characteristics, sustained GDF release, and cytocompatibility within the concentrations evaluated. Across multiple viral and bacterial particulate antigen systems, GDF-containing formulations increased nitric oxide production, autophagosome-associated fluorescence, and the expression of dendritic cell activation-associated surface markers in DC2.4 cells, with several responses comparable to those observed with Alum and AddaVax. These findings support further investigation of the cellular pathways underlying GDF-mediated immune activation and, importantly, in vivo studies to determine whether these in vitro responses translate into antigen-specific adaptive immunity.

## Figures and Tables

**Table 1:** In vitro treatment groups and corresponding doses. All microparticle formulations were suspended in complete cell-culture medium before administration to the cells.

Treatment Groups	Formulation	Microparticle Amount Administered per Well
Adjuvant/GDF-only controls	GDF microparticles	100 µg
	AddaVax microparticles	100 µg
	Alum microparticles	100 µg
H3N2 Influenza MPs	H3N2 MPs alone	100 µg
	GDF MPs + H3N2 MPs	50 µg + 100 µg
	AddaVax MPs + H3N2 MPs	50 µg + 100 µg
	Alum MPs + H3N2 MPs	50 µg + 100 µg
	AddaVax MPs + GDF MPs + H3N2 MPs	25 µg + 25 µg + 100 µg
	Alum MPs + GDF MPs + H3N2 MPs	25 µg + 25 µg + 100 µg
	Addavax MPs + Alum MPs + H3N2 MPs	25 µg + 25 µg + 100 µg
Zika MPs	Zika MPs alone	100 µg
	GDF MPs + Zika MPs	50 µg + 100 µg
	AddaVax MPs + Zika MPs	50 µg + 100 µg
	Alum MPs + Zika MPs	50 µg + 100 µg
	Alum MPs + GDF MPs + Zika MPs	25 µg + 25 µg + 100 µg
	AddaVax MPs + GDF MPs + Zika MPs	25 µg + 25 µg + 100 µg
	AddaVax MPs + Alum MPs + Zika MPs	25 µg + 25 µg + 100 µg
SARS-CoV-2 Omicron MPs	SARS-CoV-2 MPs alone	100 µg
	GDF MPs + Omicron MPs	50 µg + 100 µg
	AddaVax MPs + Omicron MPs	50 µg + 100 µg
	Alum MPs + Omicron MPs	50 µg + 100 µg
	Alum MPs + GDF MPs + Omicron MPs	25 µg + 25 µg + 100 µg
	AddaVax MPs + GDF MPs + Omicron MPs	25 µg + 25 µg + 100 µg
	AddaVax MPs + Alum MPs + Omicron MPs	25 µg + 25 µg + 100 µg
Measles MPs	Measles MPs alone	100 µg
	GDF MPs + Measles MPs	50 µg + 100 µg
	AddaVax MPs + Measles MPs	50 µg + 100 µg
	Alum MPs + Measles MPs	50 µg + 100 µg
	Alum MPs + GDF MPs + Measles MPs	25 µg + 25 µg + 100 µg
	AddaVax MPs + GDF MPs + Measles MPs	25 µg + 25 µg + 100 µg
	AddaVax MPs + Alum MPs + Measles MPs	25 µg + 25 µg + 100 µg
Gonorrhea MPs	Gonorrhea MPs alone	100 µg
	GDF MPs + Gonorrhea MPs	50 µg + 100 µg
	AddaVax MPs + Gonorrhea MPs	50 µg + 100 µg
	Alum MPs + Gonorrhea MPs	50 µg + 100 µg
	AddaVax MPs + GDF MPs + Gonorrhea MPs	25 µg + 25 µg + 100 µg
	Alum MPs + GDF MPs + Gonorrhea MPs	25 µg + 25 µg + 100 µg
	Addavax MPs + Alum MPs + Gonorrhea MPs	25 µg + 25 µg + 100 µg

**Table 2:** Physicochemical characterization of Guanine-α-D-Fructose-loaded PLGA microparticles, including particle size, polydispersity index, yield recovery, zeta potential, and entrapment efficiency.

Measurement Category	Observed Outcome
Hydrodynamic diameter (μm)	1.436 ± 0.049 μm
Polydispersity Index	0.21 ± 0.031
Microparticle Yield Recovery	94% ± 3%
Zeta Potential (mV)	−14.8 ± 0.7
Entrapment Efficiency	86.4% ± 6%

**Table 3:** Coefficient of determination (R^2^) evaluation for kinetic models describing Guanine-α-D-Fructose release from PLGA microparticles.

Model	R^2^
Higuchi	0.9234
First-order	0.8495
Korsmeyer-Peppas	0.9683
Zero Order	0.7761

## Data Availability

Data will be available on request.
